# Individualized mobile health interventions for cardiovascular event prevention in patients with coronary heart disease: study protocol for the iCARE randomized controlled trial

**DOI:** 10.1186/s12872-021-02153-9

**Published:** 2021-07-13

**Authors:** Yuling Chen, Meihua Ji, Ying Wu, Ying Deng, Fangqin Wu, Yating Lu

**Affiliations:** 1 School of Nursing, Capital Medical University, 10 You-an-men Wai Xi-tou-tiao, Feng-Tai District, Beijing, 100069 China; 2 The fourth Ward of Coronary Heart Disease Center, Emergency Coronary Ward, Beijing Anzhen Hospital, Capital Medical University, 2 Anzhen Road, Chaoyang District, Beijing, 100029 China; 3 Cardiac Center, Beijing Chao-Yang Hospital, Capital Medical University, 8 Gongti South Road, Beijing, 100020 China; 4 Advanced Innovation Center for Human Brain Protection, Capital Medical University, 119 South Fourth Ring West Road, Feng-Tai District, Beijing, China

**Keywords:** Mobile health, Health behavior, Medication adherence, Cardiovascular events, Coronary heart disease, Nursing

## Abstract

**Background:**

Mobile health-based individualized interventions have shown potential effects in managing cardiovascular risk factors. This study aims to assess whether or not mHealth based individualized interventions delivered by an Individualized Cardiovascular Application system for Risk Elimination (*iCARE*) could reduce the incidence of major cardiovascular events in individuals with coronary heart disease.

**Methods:**

This study is a large-scale, multi-center, parallel-group, open-label, randomized controlled clinical trial. This study will be conducted from September 2019 to December 2025. A total of 2820 patients with coronary heart disease will be recruited from two clinical sites and equally randomized into three groups: the intervention group and two control groups. All participants will be informed of six-time points (at 1, 3, 6, 12, 24, and 36 months after discharge) for follow-up visits. Over a course of 36 months, patients who are randomized to the intervention arm will receive individualized interventions delivered by a fully functional *iCARE* that using various visualization methods such as comics, videos, pictures, text to provide individualized interventions in addition to standard care. Patients randomized to control group 1 will receive interventions delivered by a modified *iCARE* that only presented in text in addition to routine care. Control group 2 will only receive routine care. The primary outcome is the incidence of major cardiovascular events within 3 years of discharge. Main secondary outcomes include changes in health behaviors, medication adherence, and cardiovascular health score.

**Discussion:**

If the *iCARE* trial indeed demonstrates positive effects on patients with coronary heart disease, it will provide empirical evidence for supporting secondary preventive care in this population. Results will inform the design of future research focused on mHealth-based, theory-driven, intelligent, and individualized interventions for cardiovascular risk management.

**Trial registration:**

Trial registered 24th December 2016 with the Chinese Clinical Trial Registry (ChiCTR-INR-16010242). URL: http://www.chictr.org.cn/showproj.aspx?proj=17398.

**Supplementary Information:**

The online version contains supplementary material available at 10.1186/s12872-021-02153-9.

## Background

Cardiovascular disease (CVD) remains a leading cause of death worldwide including China, with about 50% of the deaths from coronary heart disease (CHD) [1, 2]. Each year, CHD causes an estimation of 7.3 million deaths globally, with a mortality rate of 115 per 10,000 reported from China [1, 2]. Individuals diagnosed with CHD are at high risk in developing major cardiovascular events (MACEs) following discharge [3], with incidences ranging from 6 to 25% [4, 5]. As shown in previous studies, the occurrence of MACEs for patients with CHD is associated with poor health outcomes, such as increased long-term mortality, reduced quality of life, increased health care costs and caregiver burden [6].

Identified cardiovascular risk factors for CHD and MACEs, such as unhealthy behaviors (including unhealthy diet, inadequate physical activity, smoking), elevated metabolic indicators (high cholesterol, high blood pressure, high blood sugar levels) and medication nonadherence, are highly modifiable [7]. According to the American Heart Association, promotion of ideal cardiovascular health should include the combination of ideal health behaviors and ideal metabolic indicators, which illustrates a greater impact on reducing incident MACEs than any single cardiovascular risk factor [8–10]. Addressing cardiovascular health in disease management was associated with a 68% and 45% reduction of incident MACEs and CHD mortality, respectively [5, 11, 12]. However, a low prevalence of ideal cardiovascular health was identified among patients with CHD, despite interventions in facilitating cardiovascular health have been developed and implemented [13–16]. Studies from Europe, the United States, and China showed that, about 40% to 80% of the patients with CHD were not adherent to health behaviors and medication treatment [12, 15–17].

Lack of individualized real-time feedback was identified as one of the main barriers preventing patients from adapting strategies for health behaviors and medication adherence [18]. A recent meta-analysis found that individualization was a possible approach for adults to effectively reduce weight, waist circumference, and blood pressure [19]. The concept of individualization is referred to the content of the intervention being created based on individuals’ existing behaviors, stages of behavior change, preferences, barriers, and other recognizable features [19]. However, despite individualized strategies were included in traditional interventions through one-on-one format, such approach was criticized as time consuming with extensive use of healthcare resources [14, 20, 21]. This greatly limited the implementational scalability of individualized interventions in promoting cardiovascular health [20, 21].

In overcoming the existing drawbacks of traditional interventions for facilitating adherence to health behaviors and medication, mobile health (mHealth) based interventions were brought into play, which allowed cost-effective delivery of individualized interventions and real-time feedback [22, 23]. However, inconsistent findings and suboptimal effect on facilitating adherence to health behavior and medication have been reported [24–26], with evidence on the MACEs reduction remained unconvincing [24–26].

Several barriers may have prevented the effect of mHealth interventions from achieving their optimal results. Firstly, the interventions delivered by existing mobile tools were mostly simple, and they were not actually reflecting individualized contents with real-time feedback [27, 28]. Secondly, the effect of individualized mHealth-based interventions for facilitating adherence has not been well established in large-scale and long-term randomized control trials [29, 30]. Thirdly, only limited number of mHealth based interventions addressed multiple CHD related risk factors, with about 80% only focused on one of the health behaviors, such as diet and smoking [24, 31]. Fourthly, a theory-driven approach was often lacking in designing the mHealth interventions [28, 32], with limited emphasis on evidence-based content [33]. Lastly, text-messaging was the most commonly used method for delivering mHealth-based interventions [29]. It was criticized that text-messaging might be unattractive to engage patients in the interventional activities over a longer period [34]. Interestingly, application of visual contents such as videos, comics, and pictures are better fitted for meeting patients’ preferences, therefore can possibly improve the attractiveness of mHealth interventions [35]. A recent study on visualization of asymptomatic atherosclerotic disease for optimum cardiovascular prevention provides evidence to support the contributory role of pictorial presentation on CVD prevention [36]. However, whether or not mHealth interventions delivered via visual modes can effectively promote health behavior change and medication adherence in CHD patients, therefore leading to a reduced incidence of MACEs, is unclear [36].

### Preliminary work: iCARE system

In order to facilitate ideal cardiovascular health therefore reducing the incidence of MACEs in patients with CHD, we developed an **I**ndividualized **C**ardiovascular **A**pplication system for **R**isk **E**limination (*iCARE*). The development process and the functions of the *iCARE* system were described in our preliminary work (Additional file 1). In summary, the *iCARE* system was developed based on a user-centered approach as suggested by previous research [37]. We incorporated a set of IF–THEN algorithm triggering interventions to ensure patients receiving individualized recommendations and real-time feedbacks for facilitating adherence to health behaviors and medications. The interventions of the *iCARE* were developed based on the intervention mapping framework and the Contemplation-Action-Maintenance (CAM) behavior change model for CHD patients [38]. The needs and preferences of patients with CHD, as well as professional opinions of nurses and clinicians who are specialized in CHD were fully considered during the development process. The *iCARE* system includes a patient app, a care-provider app for healthcare professionals, and a cloud platform. The main functional components of the *iCARE* system are displayed in Additional file 1.

This study protocol is aimed to describe the implementational plan for a randomized controlled clinical trial in which the effect of the individualized, visualized, comprehensive interventions delivered by *iCARE* on MACEs reduction for patients with CHD will be evaluated. Meanwhile, the study plan for testing the effects of the *iCARE* on facilitating adherence to recommended health behaviors (including healthy diet, regular physical activity, and smoking cessation) and medications will also be described.

## Methods

### Study design

The *iCARE* study is a multicenter, open-labeled randomized controlled trial with three parallel groups conducted among patients with CHD. The *iCARE* trial has been registered at Chinese Clinical Trial Registry in December 2016 (ChiCTR-INR-16010242) prior to participants’ recruitment. This trial will be conducted in accordance with the SPIRIT 2013 statement [39] and the Consolidated Standards of Reporting Trials (CONSORT) for Web-based and Mobile Health Interventions [40]. The results will be reported according to the CONSORT guidelines [41]. This study is approved by the Institutional Review Committee of the Capital Medical University (Approval No. 2015SY45) and the study hospitals (Beijing An-Zhen Hospital, Approval No. 2015030; Beijing Chao-Yang Hospital, Approval No. 20211224). Written informed consent will be obtained from all patients who meet the inclusion criteria and are willing to participate before randomization.

### Settings and participants

Eligible patients will be recruited from three cardiac wards and one emergency ward in two university affiliated hospitals. All participants will be followed up for a total of three years (with six time-points for data collection). Patients will be included in the study if they: (a) are 18 years or older; (b) have a documented diagnosis of CHD including acute myocardial infarction (AMI), acute coronary syndrome (ACS), or undertake a percutaneous coronary intervention (PCI) either as emergency or elective procedures; (c) have at least one smartphone with Android system and use it daily, and (d) agree to participate. We choose smart phones with android system to install the *iCARE* as they are more popular and affordable in China [31].

The exclusion criteria include: patients who are (1) with a history of psychiatric and neurological disorders; (2) unable to speak or understand Mandarin; (3) illiterate; (4) with impaired bilateral hearing, or visual impairment which limits the use of smartphone; (5) with contraindications or severe physical disabilities that limit patients from participation; (6) with severe medical conditions; and (7) participating in other ongoing clinical trials or prospective cohort studies. Additional exclusion criteria will be applied if patients are not able to understand the procedure and the aims of the study after full explanation, or not able to fill in the questionnaires during the baseline visits.

### Recruitment, screening, and baseline visits

To identify eligible patients, a targeted review of patients’ medical record based on the inclusion and exclusive criteria will be carried out, with referrals from nurses or clinicians within the study sites. Identified patients with CHD will participate in a formal screening session and have a baseline visit in the hospital. During the formal screening session, we will ask patients to complete an initial screening assessment which includes age, educational background, vision, hearing, diagnosis, heart function grade, etc. Patients who meet the study criteria will be asked to sign a written informed consent before attending the baseline visit. Participants’ baseline data will be collected via the *iCARE* care-provider app and patient app within one week of enrollment, which includes patients’ general characteristics, health behaviors (daily diet, physical activities, smoking status) and medication adherence in the past 30 days before admission, as well as their blood pressure, blood glucose, blood lipid levels during hospitalization.

### Randomization and blinding

In this proposed study, three versions of *iCARE* apps will be installed on participant’s smart phone based on their group assignment (Additional file 1). Study investigators will assist participants in each group to download, register and log into the designated *iCARE* app:

1. *iCARE*-1: is fully functional with individualized interventions delivered via multiple visual displays such as comics, videos, pictures, and text to address all the factors in the CAM model plus routine care;
2. *iCARE*-2: is fully functional but all interventions are delivered only via text which did not address all factors in the CAM model plus routine care;
3. *iCARE*-3: has limited function which only allows patients for daily data entry.

After baseline data collection, participants will be randomized to one of the three groups: the intervention group (*iCARE*-1 plus routine care), the control group 1 (*iCARE*-2 plus routine care), or the control group 2 (*iCARE*-3 plus routine care), following stratified block randomization. The randomization will be computer-generated by a statistician who will not have any active involvement in the study. The block size will be set at 15 with the allocation ratio set at 1:1:1 to ensure balance among three study groups. Participants will be stratified by 4 wards and their smoking status. The randomization assignment will be generated randomly, numbered consecutively, and will be kept in a sealed, opaque envelope. Patient assignment will be revealed to study researchers only after participants completed their baseline visit. This proposed study is an open-labeled trial as participants are fully aware of their intervention allocation, and the group assignments were known to study investigators. However, researchers who perform data analysis, and clinical staff who conduct laboratory testing will be unaware of the group assignment.

## Intervention

### Routine care for all participants

All participants will receive routine care designated for discharged patients at each study site, in which an information leaflet about the benefits of adhering to health behaviors and medication, and the time points for follow-up visits will be distributed to all patients. In addition to the baseline questionnaire (T0) completed before participants are discharged from hospitals, all participants will need to complete a set of electronic questionnaires on the *iCARE* patient app and answer questions by telephone at 1 (T1), 3 (T2), 6 (T3), 12 (T4), 24 (T5), and 36 (T6) months after they are discharged from hospitals. Participants’ responses to these questionnaires will be used to evaluate the study outcomes. Each participant will receive 100 RMB (equivalent to 14 USD) to reimburse any costs (time, internets services, etc.) associated with participation of the trial. All participants will receive a wrist watch which has functions of monitoring their heart rate and counting daily steps.

### Intervention group

Patients randomized to the intervention arm will receive individualized care delivered via the *iCARE*-1 plus routine care over a course of 36 months. After participants complete the baseline assessment, they will receive a personalized initial health report which describes their medical and nursing diagnosis, modifiable unhealthy behaviors and health factors, along with established goals and action plans for risk reduction. They can also access individualized interventions with visual formats (such as animation, video, cartoon, and picture) for facilitating their adherence to health behaviors and medications. The interventions of *iCARE*-1 were developed based on existing evidence-based health behavior change strategies, and the mediators and moderators identified in the CAM model [42]. Participants in this group can enter their health-related data into the *iCARE*-1 by manual entry. These include diet, number of cigarettes smoked, level of physical activity, lipid profile, fasting blood glucose, hemoglobin A1c, blood pressure, heart rate, and weight. Information on participants’ heart rates and daily steps can also be synchronized through a smart watch. All data will be uploaded to the cloud platform and the tele-monitoring system for analysis. Instant and individualized feedback and tailored recommendations will be automatically sent to the care-provider app based on the built-in algorithms for verification by cardiovascular nurses. Verified interventions will be automatically sent to participants on *iCARE*-1. The described study design will not change once recruitment begins. However, participants are informed of options on stopping receiving the interventions at any time, during the consent process.

### Control groups

*Control group 1* participants in control group 1 will receive routine care plus interventions via the *iCARE*-2 in which all interventions are delivered only via text format.

*Control Group 2* participants in control group 2 will receive routine care and be asked to install the *iCARE*-3 before they are discharged. The *iCARE*-3 allows us to collect patients’ baseline and follow-up information through the app therefore to minimize information bias in estimation of the intervention effect.

### Outcomes

#### Primary outcome

The primary outcome of this study is the occurrence of MACEs, a binary variable based on the occurrence of AMI/ACS/stable angina, coronary revascularization, stroke, hospitalized heart failure, or death attributable to any cardiovascular disease [3], during the study period. Methods to ascertain MACEs are described in the Additional file 2. Based on the criteria for MACEs, information on the primary outcome will be systematically obtained through follow-up interviews at 1, 3, 6, 12, 24, and 36 months, and confirmed by reviewing patients’ medical records. If a patient dies during follow-up, this information will be obtained directly from patient’s family or confirmed by physician. All investigators in this study will attend rigorous training on data collection and research protocol. Table 1 presents all outcome measures and the time points when they will be assessed.

**Table 1:** Standard Protocol Items (SPIRIT): details on the scheduled activities related to patient enrollment, interventions, and study variables at different time points

	Study period							
	Enrollment	Allocation	Post allocation					Closeout
Time point	−T_1_	T_0_	T_1_	T_2_	T_3_	T_4_	T_5_	T_6_
*Enrollment*								
Eligibility screen	X							
Informed consent	X							
Allocation		X						
Interventions		X	X	X	X	X	X	X
Assessments								
Major cardiovascular events			X	X	X	X	X	X
*Health behaviors*								
Smoking status		X	X	X	X	X	X	X
Dietary intake		X	X	X	X	X	X	X
Physical activity		X	X	X	X	X	X	X
Medication adherence		X	X	X	X	X	X	X
*Anthropometry parameters*								
Weight		X	X	X	X	X	X	X
Body mass index		X	X	X	X	X	X	X
Waist circumference		X	X	X	X	X	X	X
*Biochemical parameters*								
Total cholesterol		X		X	X	X	X	X
Low-density lipoprotein cholesterol		X		X	X	X	X	X
High-density lipoprotein cholesterol		X		X	X	X	X	X
Fasting blood glucose		X		X	X	X	X	X
Hemoglobin A1c		X		X	X	X	X	X
Systolic blood pressure		X		X	X	X	X	X
Diastolic blood pressure		X		X	X	X	X	X
10-year Framingham risk scores of cardiovascular risks		X		X	X	X	X	X
All-cause mortality			X	X	X	X	X	X
All-cause readmission			X	X	X	X	X	X
Frequency of medical treatment due to CHD			X	X	X	X	X	X
Patients’ engagement in the intervention			X	X	X	X	X	X
*Mediators and moderators of behavior change*^a^								
Risk perception		X			X	X	X	X
Outcome expectation		X			X	X	X	X
Action planning		X			X	X	X	X
Coping planning		X			X	X	X	X
Behavioral enjoyment		X			X	X	X	X
Social support		X			X	X	X	X
Self-efficacy		X			X	X	X	X
Effectiveness perception		X			X	X	X	X
Intention		X			X	X	X	X
Motivation		X			X	X	X	X
Volition		X			X	X	X	X
Stages of behavior change^a^		X			X	X	X	X
Knowledge of CHD		X			X	X	X	X
Perceived importance of health behaviors		X			X	X	X	X

CHD, coronary heart disease. −T1: Start point for patient recruitment; T0: Baseline; T_1_: 1 month; T_2_: 3 months; T_3_: 6 months; T_4_: 12 months; T_5_: 24 months; T_6_: 36 months

^a^The stages of behavior change, mediators and moderators of behavior change will be assessed dynamically

#### Secondary outcomes

The secondary outcomes of the study are summarized in Table 2 (the definitions and detailed measuring methods of all secondary outcomes are listed in Additional file 2). A copy of questionnaires for the study was shown in Additional file 3. Main secondary outcomes include changes in health behaviors, medication adherence, and cardiovascular health score:

1. Healthy diet: dietary intake will be assessed by a short food frequency questionnaire (FFQ) which was developed according to the Chinese Guidelines on Cardiovascular Disease Prevention and previous study [43, 44]. The FFQ includes the amount and frequency of intakes on grains, vegetables, fruits, fish, and meat (poultry) in the past month [44]. The dietary pattern is categorized into three levels (ideal healthy diet, intermediate healthy diet, and poor diet) based on the recommended dietary goals from the European Guidelines on Cardiovascular Disease Prevention [7] and Chinese Expert Consensus on CHD Secondary Prevention [43].
2. Physical activity: participants’ physical activity will be assessed by the simplified Chinese Version of the International Physical Activity Questionnaire (IPAQ), which was reported with acceptable test–retest reliability with intraclass correlation coefficients at 0.57–0.73 among Chinese population [45]. The IPAQ evaluates the intensity, frequency, and time spent on leisurely activities and occupational tasks in the past week. The scores of IPAQ will be converted to metabolic equivalents of task (METs) for each activity level based on the criteria from European Guidelines on Cardiovascular Disease Prevention [7]. Low physical activity refers to less than 600 METs/min/week [46].
3. Smoking: participants’ smoking status will be assessed by a 32-item smoking status questionnaire which was adopted from previous research [44]. This questionnaire evaluates the frequency of smoking, number (current and past) of cigarettes smoked in the past 30 days, age started smoking among participants. Participants will be categorized as current smoker, former smoker, and nonsmoker. In addition, participants’ level of nicotine dependence will also be assessed using the 6-item Chinese version of the Fagerström Test for Nicotine Dependence (FTND) which has been documented to be a useful instrument for evaluating nicotine dependence in Chinese adults [47, 48].
4. Adherence to secondary preventive medications: Medication adherence will be measured by the self-reported 8-item Morisky Medication Adherence Scale (MMAS-8) based on participant’s prescribed medications [49, 50]. The validated Chinese version of the MMAS-8 (C-MMAS-8) has a good internal consistency (Cronbach’s α = 0.77) and test–retest reliability [51], with the total score ranging from 0 to 8, and the higher the score, the better the medication adherence. The C-MMAS-8 includes 7 items with yes (“1”) or no (“0”) responses and one item on a 5-point Likert scale (0.2 to 1). Participants’ prescribed medications will be uploaded to the *iCARE* patients’ app before patients are discharged. Therefore, for patients in the intervention group and control group 1, the medication adherence will be also calculated by the *iCARE* patients app.
5. Cardiovascular health: the American Heart Association defines the ideal cardiovascular health as simultaneous presence of four ideal health behaviors and three ideal metabolic indicators [8]. The four ideal health behaviors include nonsmoking, body mass index (BMI) < 25 kg/m^2^, physical activity at targeted level, and dietary consumption being consistent with current recommended guidelines. The three ideal metabolic indicators are identified as clinical parameters for blood pressure < 120/80 mm Hg, total cholesterol < 5.17 mmol/L (200 mg/dL) and fasting glucose < 5.60 mmol/L (100 mg/dL). Each component of the cardiovascular health metrics will be dichotomized as 1 (ideal status) or 0 (intermediate or poor status), and the cumulative number of ideal cardiovascular health metrics will be calculated to reflect patients’ overall cardiovascular health, with a possible range from 0 to 7 points [45].

**Table 2:** The secondary outcomes and related measurements assessed in the iCARE study

Secondary outcomes	Measurements or tools
*Health behaviors*	
Dietary intake	A Short Food Frequency Questionnaire (FFQ) which was developed for this study according to the Chinese Guidelines on Cardiovascular Disease Prevention [43, 44]
Physical activity	The Simplified Chinese Version of the International Physical Activity Questionnaire (IPAQ) [45]
Smoking status	A 32-item Smoking Status Questionnaire which was developed for this study according to smoking history questionnaire from Lv et al. [44]
Medication adherence	A validated 8-item Chinese version of Morisky Medication Adherence Scale (C-MMAS-8) ^a^ [50, 51]
Cardiovascular health	American Heart Association Cardiovascular Health Score [8]
Anthropometry parameters	A Baseline Assessment Questionnaire (A) which was developed for this study
Biochemical parameters	Collected through follow-up visits with a Follow-up Assessment Questionnaire which was developed for this study and confirmed by reviewing medical records
All-cause mortality	Collected through follow-up visits with a Follow-up Assessment Questionnaire which was developed for this study and confirmed by reviewing medical records
All-cause readmission	Collected through follow-up visits with a Follow-up Assessment Questionnaire which was developed for this study and confirmed by reviewing medical records
Frequency of medical treatment	Collected through follow-up visits with a Follow-up Assessment Questionnaire which was developed for this study and confirmed by reviewing medical records
Patients’ engagement in the intervention	Data derived from iCARE platform: (1) the number of screens patients visited, (2) the frequency in uploading data, and (3) the number of interventions they accessed while using the patient application
Cardiovascular risks	10-year Framingham risk scores of cardiovascular risks tool [59]
*Mediators and moderators*	
Risk perception	A 4-item Risk Perception on Cardiovascular Risks Scale which was adapted from Renner and Schwarzer [60]
Outcome expectation	Outcome Expectation of Healthy Diet Scale, Outcome Expectation of Regular Exercise Scale, and Outcome Expectation of Quitting Smoking were from Renner and Schwarzer [60]. Outcome Expectation of Medication Management Scale was developed for this study
Action planning	Action Planning of Healthy Diet Scale, Action Planning of Regular Exercise Scale, and Action Planning of Quitting Smoking Scale were from Renner and Schwarzer [60]. Action Planning of Medication Management Scale was developed for this study
Coping planning	Coping Planning of Healthy Diet Scale, Coping Planning of Regular Exercise Scale, and Coping Planning of Quitting Smoking were from Renner and Schwarzer [60]. Coping Planning of Medication Management Scale was developed for this study
Behavioral enjoyment	Enjoyment of physical activity will be assessed using the 8-item Physical Activity Enjoyment Scale [61]. Enjoyment of healthy eating, smoking cessation, and adherence to medication therapy will be assessed with the scales adapted from Physical Activity Enjoyment Scale [61]
Social support	A 12-item validated Chinese Multidimensional Scale of Perceived Social Support (MSPSS) [62]
Self-efficacy	Self-efficacy of Diet Scale, Self-efficacy of Exercise Scale, and Self-efficacy of Quitting Smoking were from Renner and Schwarzer [60]. Self-efficacy of Medication Management Scale was developed for this study
Effectiveness perception	A 5-item Visual Analogue Scale (ranging from 0 to 10) of effectiveness perception of changes in blood pressure, lipid level, blood glucose, weight, and vascular plaque, which was developed for this study
Intention	A Visual Analogue Scale (ranging from 0 to 10) of intention to change unhealthy diet, physical inactivity, smoking was from Renner and Schwarzer [60]. Intention to change medication nonadherence scale was developed for this study
Motivation	A 4-item Visual Analogue Scale (ranging from 0 to 10) of motivation to change unhealthy diet, physical inactivity, smoking, and medication nonadherence, which was developed for this study according to Visual Analog Scale for motivation to quit from Vitor de Souza Brangioni et al. [63]
Volition	A 4-item Visual Analogue Scale (ranging from 0 to 10) of volition to maintain healthy behaviors and medication adherence, which was developed for this study
Stages of behavior change	A 4-item Stages of Behavior Change Scale [64]
Knowledge of CHD	Coronary artery disease education questionnaire (CADE-QII) [65]
Perceived importance of health behaviors	A Visual Analogue Scale (ranging from 0 to 10) of patients’ perceived importance of health behaviors [66]

CHD: Coronary heart disease

^a^The MMAS (8-item) content, name, and trademarks are protected by US copyright and trademark laws. Permission for use of the scale and its coding is required. A license agreement is available from Donald E. Morisky, ScD, ScM,MSPH, 14725 NE 20th St Bellevue, WA 98007, USA; dmorisky@gmail.com

### Sample size calculation and power analysis

Based on previous studies [3, 4, 52], we anticipated that the 3-year incidence rate for cardiovascular events is 10% for patient with CHD. We estimated that a total of 2256 participants (752 per group) would provide 80% power to detect a 40% relative reduction [53] in MACEs (the incidence of MACEs is 10% for the control group and 6% for the intervention group, hazard ratio is 0.6), with 2-sided testing and alpha set at 0.05, and following a Cox proportional hazards regression method. According to previous studies [34, 54] and based on our pilot study, we anticipated a maximum attrition rate of 20% among all enrolled patients. Therefore, a total sample size of 2820 participants (940 per group) are needed in the present study.

### Data analyses

For baseline characteristics, categorical variables will be described as frequencies and proportions; continuous variables will be described as means and standard deviations, or medians and interquartile ranges where appropriate. Differences in baseline characteristics between the control and intervention groups will be detected using ANOVA test (continuous variables) or χ^2^ test (categorical variables) where appropriate.

Comparison of between group differences on the primary outcome will be performed according to both the intention-to-treat and the per-protocol principles. Simple χ^2^ analyses will be used to evaluate the main effect of the intervention on the MACEs at the end of the intervention period. Cox proportional hazards regression (with time to first event) will be used to estimate hazard ratios (HRs) comparing MACEs among three groups, by considering study group as an independent variable adjusted for covariates that are used for stratification (i.e., study sites, smoking status). The HRs and 95% *CI* will be calculated. Significant differences will be reported at the alpha level of 0.05 (two-sided).

All analyses related to secondary outcomes of the study are exploratory. The results of the analysis will be used to clarify the interpretation of the results related to the primary analysis, which include: changes in the proportion of adherence to health behaviors and medication, changes in anthropometry and biochemical parameters, etc. The mixed models and multilevel logistic regression models will be used to assess the differences in secondary outcomes between groups. Meanwhile, we will also explore whether the effects of intervention on the primary and secondary outcomes are consistent or not across subgroups of interest. Sensitive analyses will also be conducted.

### Quality control

Because the *iCARE* patient app is not listed in the app store yet, patients cannot download and install the app by themselves. Therefore, those in the control groups are not accessible to the apps with full functions. Meanwhile, patients in the control groups are automatically putting on a waiting-list which offers the choice to allow them install the *iCARE*-1 at the end of the study. However, this study cannot avoid communications among patients who are discharged from the same hospital units. Whether or not the communication occurred will be determined by asking patients during their follow up. Should any contamination occurred, the patient will be eliminated.

### Process evaluation

Upon completion of the current study, process evaluation will be conducted to evaluate the feasibility of the mHealth intervention as recommended by researchers [55, 56]. The fidelity of intervention delivery, the exposure to the intervention, and feedback based on user’s experiences both from patients and nurses will be addressed in the process evaluation [56]. We will also ask patients to report on the reasons of non-adherent to health behaviors and medications during follow-up.

### Safety monitoring and event adjudication

Patients’ safety will be carefully monitored by a Data and Safety Monitoring Board from the *iCARE* research team. When there are adverse events occurred during the study, the time, place and cause of adverse events will be recorded. Potentially, vulnerable patients of the study are patients older than 75 years old and those with osteoporosis. Primary and secondary outcomes will be adjudicated by consensus among three investigators who are masked to the group assignments and results.

### Regulatory, data management and monitoring

The study will be conducted in accordance with the Good Clinical Practice, an international ethical and scientific standard for the design, conduct, performance, monitoring, auditing, recording, analysis, and reporting of clinical trial. Primary responsibility for data monitoring will be assumed by the principal investigator, who will oversee the integrity, security, and sharing of data, and take the primary responsibility for ensuring that the design of the study meets appropriate standards. Researchers will clean data collected through the *iCARE* system weekly, verify completeness and accuracy of the data.

### Confidentiality

Participants’ personal data will be replaced with research identification codes; files containing electronic data are password-protected and encrypted. The results will be disseminated in a deidentified manner for protecting the privacy and confidentiality of participants.

### Study status and recruitment

The participant recruitment process has started since September 2019 and is in progress. As recruitment is planned to be finalized in December 2022 and patients will be followed for 3 year, the final study results will be first available by December, 2025. Therefore, data cleaning or analyses are not yet started at the time of submission.

## Discussion

Individualized mHealth-based interventions represented a promising solution for facilitating adherence to health behaviors and medications, therefore, may decrease the burden of MACEs among patients with CHD. Although numerous studies have been implemented with individualized mHealth-based interventions, the long-term effects of such interventions on the reductions of MACEs have not been well established [24, 29, 30]. The influence of individualized mHealth-based interventions on MACEs was also not determined due to small sample sizes in previous studies [29, 30]. In the present study, the long-term effects of *iCARE* interventions on MACEs reduction in patients with CHD will be determined. In addition, previous study indicated that the challenges of CHD prevention were the maintenance of healthy behaviors [57]. The interventions of *iCARE* study were structured following the CAM model which focused on the moderators and mediators of the action and maintenance stages of behavioral change among patients with CHD [42]. The utilization of theory-driven interventions in the *iCARE* study may improve the likelihood of maintaining adherence to health behaviors [58]. If our *iCARE* study indeed demonstrates positive effects on patients with CHD, it will provide empirical evidence for supporting secondary preventive care in this population. Results will inform the design of future research focused on mHealth-based, theory-driven, intelligent, and individualized interventions for cardiovascular risk management.

## Supplementary Information

**Additional file 1**. The components and functions of the *iCARE*.

**Additional file 2**. Definitions and instruments of administration for study variables.

**Additional file 3**. Questionnaires for the *iCARE* trial.

**Additional file 4**. SPIRIT 2013 Checklist.

## Data Availability

Data not applicable for a protocol. The results will be available to the public if necessary. The study findings will be disseminated via peer-reviewed publications and conference presentations.

## Abbreviations

ICARE: Individualized cardiovascular application system for risk elimination
ACS: Acute coronary syndrome
AMI: Acute myocardial infarction
BMI: Body mass index
CAM: Contemplation-action-maintenance
CHD: Coronary heart disease
CONSORT: Consolidated standards of reporting trials
CVD: Cardiovascular disease
FFQ: Food frequency questionnaire
FTND: Fagerström test for nicotine dependence
HRs: Hazard ratios
IPAQ: International physical activity questionnaire
MACEs: Major cardiovascular events
METs: Metabolic equivalents of task
MMAS-8: 8-Item Morisky medication adherence scale
PCI: Percutaneous coronary intervention
