# Supplementary material for: Individualized mobile health interventions for cardiovascular event prevention in patients with coronary heart disease: study protocol for the iCARE randomized controlled trial
Source: BMC Cardiovasc Disord. 2021 Jul 13;21:340. doi: 10.1186/s12872-021-02153-9 (PMC8278759; doi:10.1186/s12872-021-02153-9)
Supplement: Supplementary file 1 — Additional file 1. The components and functions of the iCARE. [file 12872_2021_2153_MOESM1_ESM.docx]

**Supplementary file 1. The components and functions of the iCARE**

| **Components** | **Users** | **Functions** |
| --- | --- | --- |
| iCARE-1 | Patients in the intervention group | Allowing patients to enter personal data related to health behaviors, prescribed medications (for adherence monitoring) and other physiological indicators; review individual health data and health report, as well as generate recommended interventional measures by the system. Interventions are delivered via multiple displays such as comics, videos, pictures, and words to address all the factors in the Contemplation-Action-Maintenance model plus routine care. |
| iCARE-2 | Patients in the control group 1 | Has full functions but all interventions are delivered only via word format. |
| iCARE-3 | Patients in the control group 2 | Has function of daily data entry only. |
| Care-provider app | Healthcare providers | Allowing healthcare providers to view patients’ health data and health report, make health assessment, confirm recommended interventional measures, and provide health consultation as necessary. |
| Cloud platform | Healthcare providers | Allowing healthcare providers with authorized access to view patients’ health behaviors, prescribed medications, and physiological indicators; create and edit intervention banks, knowledge banks, and rule banks; assign roles of users; conduct data analysis; and manage follow-up interviews. |
| Wrist watch | Patients in all groups | Monitoring heart rate and counting daily steps. |

Reference: Chen Y, Ji M, Wu Y, et al. Development and Usability Testing of an Individualized Cardiovascular Application for Risk Elimination (iCARE) for Individuals with Coronary Heart Disease. JMIR Medical Informatics. Under review. June, 2021.

**
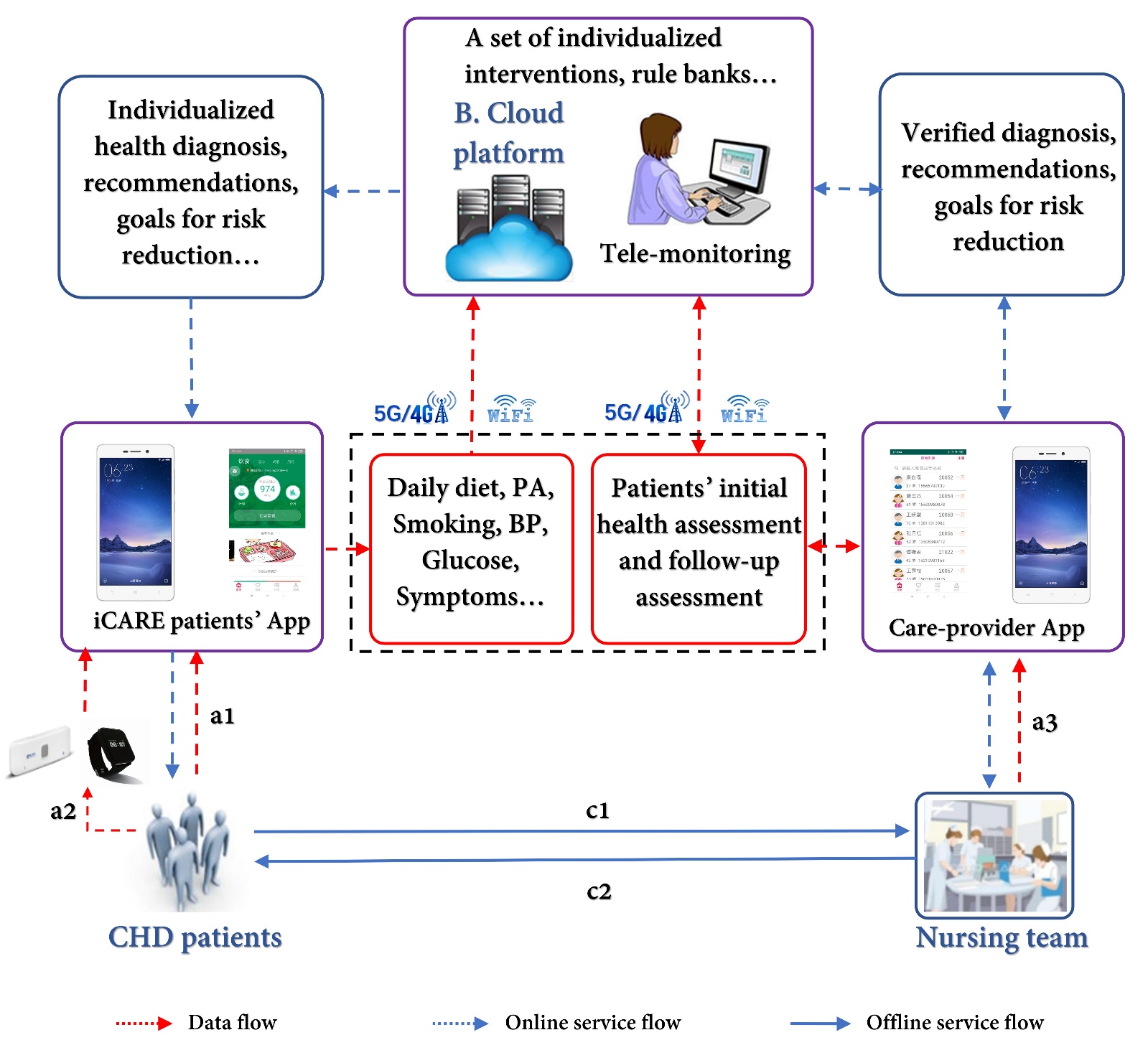
**

**The components and schematic diagram of the iCARE**

iCARE, Individualized Cardiovascular Application for Risk Elimination. CHD, coronary heart disease. a1, Initial and periodic assessment through daily monitoring via manual entry by patients. a2, Daily monitoring by wearable devices. a3, Patients’ initial assessment data, follow-up data. c1, Initial and periodic assessment by CV nurses. c2, Further evaluation, interventions, follow-up. App, application. PA, physical activity. BP, blood pressure. CHD, coronary heart disease.

Reference: Chen Y, Ji M, Wu Y, et al. Development and Usability Testing of an Individualized Cardiovascular Application for Risk Elimination (iCARE) for Individuals with Coronary Heart Disease. JMIR Medical Informatics. Under review. June, 2021.

**
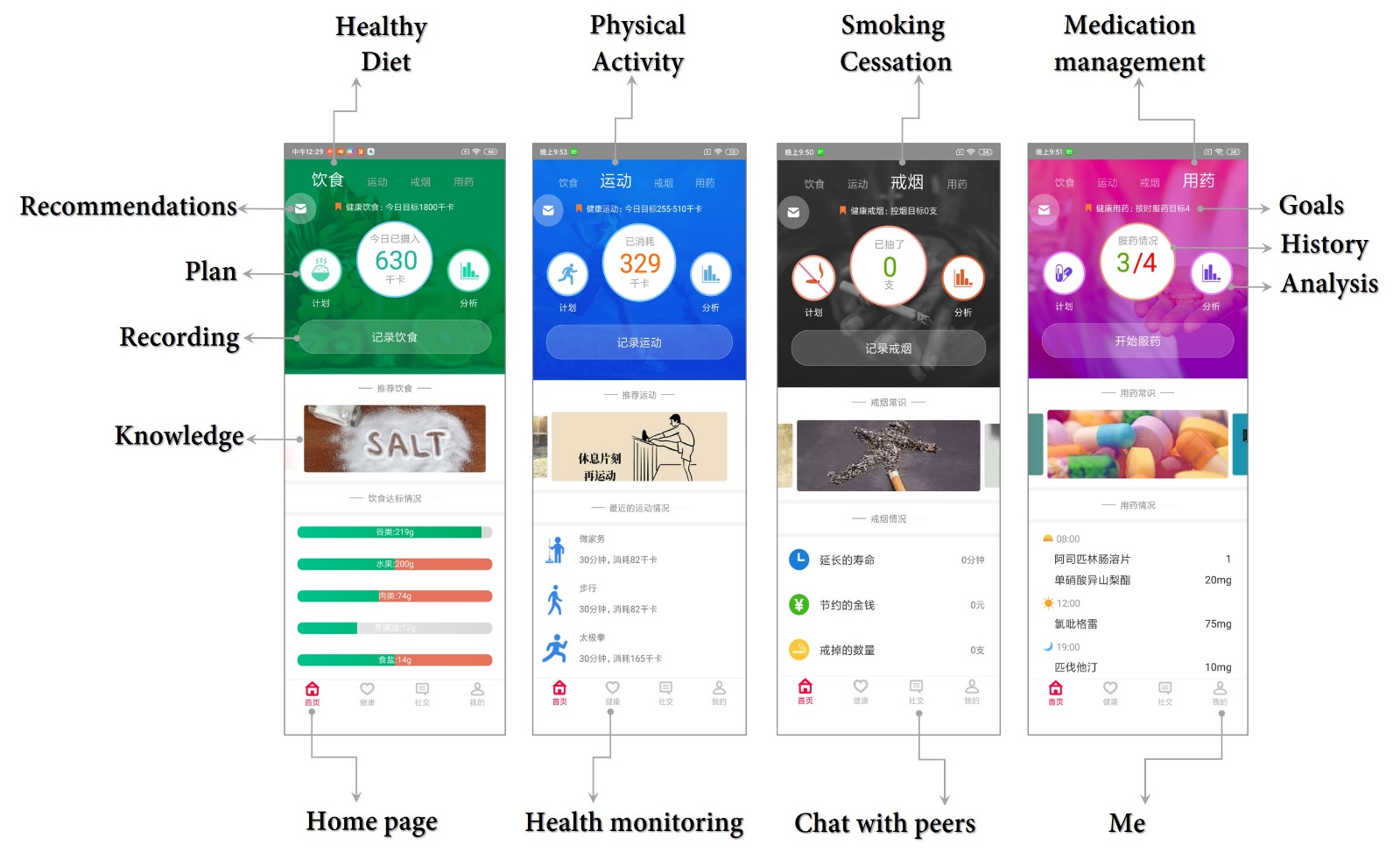
**

**The user interface of the home page of the patient app**

iCARE, Individualized Cardiovascular Application for Risk Elimination.

Reference: Chen Y, Ji M, Wu Y, et al. Development and Usability Testing of an Individualized Cardiovascular Application for Risk Elimination (iCARE) for Individuals with Coronary Heart Disease. JMIR Medical Informatics. Under review. June, 2021.
