# Supplementary material for: Individualized mobile health interventions for cardiovascular event prevention in patients with coronary heart disease: study protocol for the iCARE randomized controlled trial
Source: BMC Cardiovasc Disord. 2021 Jul 13;21:340. doi: 10.1186/s12872-021-02153-9 (PMC8278759; doi:10.1186/s12872-021-02153-9)
Supplement: Supplementary file 2 — Additional file 2. Definitions and instruments of administration for study variables. [file 12872_2021_2153_MOESM2_ESM.docx]

**Supplementary file 2.** **Definitions and instruments of administration for study variables**

1. **Primary Outcome**

| Variables | Definition | Instruments of administration |
| --- | --- | --- |
| Major cardiovascular events | The occurrence of recurrent acute myocardial infarction or acute coronary syndrome, stable angina, coronary revascularization, stroke (ischemic or hemorrhagic), hospitalized heart failure, or death attributable to any cardiovascular disease within 3-year of discharge [1].  **Recurrent acute myocardial infarction or acute coronary syndrome:** The term ***acute myocardial infarction (AMI)*** should be used when there is evidence of myocardial injury (defined as an elevation of cardiac troponin values with at least one value above the 99th percentile upper reference limit) with necrosis in a clinical setting consistent with myocardial ischemia. ***Acute coronary syndrome*** is commonly associated with three clinical manifestations: ST elevation myocardial infarction (STEMI), non-ST elevation myocardial infarction (NSTEMI), or unstable angina.  **Recurrent stable angina:** A typical presentation of ***stable angina*** is that of chest discomfort and associated symptoms precipitated by some activity (running, walking, etc.) with minimal or non-existent symptoms at rest or after administration of sublingual nitroglycerin. Symptoms typically abate several minutes after activity and recur when activity resumes.  **Coronary revascularization: *Coronary revascularization*** refers to documented coronary artery bypass graft (CABG) surgery or percutaneous transluminal coronary angioplasty (PTCA) or percutaneous coronary intervention (PCI).  **Ischemic/hemorrhagic stroke: *Ischemic stroke*** is defined as an acute symptomatic episode of focal or global neurological dysfunction caused by brain, spinal, or retinal vascular injury as a result of infarction. ***Hemorrhagic stroke*** occurs when a blood vessel in the brain breaks, leaking blood into the surrounding tissue.  **Hospitalized heart failure: *Heart failure*** is a clinical syndrome characterized by typical symptoms (e.g., breathlessness, ankle swelling and fatigue) that may be accompanied by signs (e.g., elevated jugular venous pressure, pulmonary crackles and peripheral edema) caused by a structural and/or functional cardiac abnormality, resulting in a reduced cardiac output and/ or elevated intracardiac pressures at rest or during stress. ***Hospitalized heart failure*** is defined as an event where the patient is admitted to the hospital with a primary diagnosis of heart failure and the length of stay is at least 24 hours (or extends over a calendar day if the hospital admission and discharge times are unavailable), exhibits new or worsening symptoms of heart failure on presentation, has objective evidence of new or worsening heart failure, and receives treatment (initiation or intensification) specifically for heart failure.  **Death attributable to any cardiovascular disease:** includes death due to AMI, heart failure, stroke, coronary revascularization, hospitalized unstable angina, etc. | Based on the criteria for MACEs, information on the primary outcome will be systematically obtained through follow-up interviews at 1, 3, 6, 12, 24, and 36 months, and confirmed by reviewing patients’ medical records. If a patient dies during follow-up, this information will be obtained directly from patient’s family or confirmed by physician. All investigators in this study will attend rigorous training on data collection and research protocol. |

1. **Secondary Outcomes**

| Variables | Definition | Instruments of administration |
| --- | --- | --- |
| Diet intake | For the definition of a healthy diet, we categorize the healthy diet into seven components: 1) grains ≥ 250 g/day, 2) vegetables ≥ 300 g/day (2-3 servings), 3) fruits ≥ 200 g/day (2-3 servings), 4) fish (at least once a week) , 5) meat (poultry) ≤ 75 g/day, 6) salt (from moderate to very light), and 7) cooking oil (from moderate to very light), according to the recommended diet goals of European Guidelines on Cardiovascular Disease Prevention [2] and Chinese Expert Consensus on CHD Secondary Prevention [3]. Based on Chinese Expert Consensus on CHD Secondary Prevention [3], participants’ dietary patten will be categorized into 3 types: 1) having an ideal healthy diet score (with 6-7 of these 7 dietary components), 2) having an intermediate diet score (with 3-5 of these 7 dietary components), 3) having a poor diet score (with 0 or 2 of these 7 dietary components). Having an intermediate or poor diet score is defined as unhealthy diet. | Dietary intake will be assessed by a short food frequency questionnaire (FFQ) which was developed according to the Chinese Guidelines on Cardiovascular Disease Prevention [3], and was valid to assess healthy diet in Chinese adult [4]. The FFQ includes the amount and frequency of grains, vegetables, fruits, fish, and meat (poultry) intake in the past month [4]. We will use two questions to assess participants’ preference interns of salt and cooking oil: 1) what is your preference for salt (answers may be 1-very salty, 2-a little salty, 3-moderate, 4-a little light, and 5-very light), and 2) what is your preference for cooking oil (answers may be 1-very oily, 2-a little oily, 3-moderate, 4-a little light, and 5-very light). |
| Physical activity | According to the European Guidelines on Cardiovascular Disease Prevention [2], physical activity will be classified into three levels: 1) ideal physical activity: ≥150 min/week moderate intensity or ≥75 min/week vigorous intensity or ≥150 min/week moderate and vigorous physical activity, 2) intermediate physical activity: 1 to 149 min/week moderate intensity or 1 to 74 min/week vigorous intensity or 1 to 149 min/week moderate and vigorous physical activity, and 3) poor physical activity: no physical activity. Having an intermediate or poor physical activity is defined as physical inactivity. Low physical activity refers to less than 600 METs/min/week [5]. | Physical activity level will be assessed by a simplified Chinese-character Version of the International Physical Activity Questionnaire (IPAQ) [6]. The IPAQ questionnaire has a moderate to good test-retest reliability with intraclass correlation coefficients of 0.57-0.73 [6]. The IPAQ questionnaire includes questions related to the intensity, frequency, and time spent on leisure time activities and occupational tasks in the past week. Following the IPAQ scoring procedure, data from IPAQ items is converted to scores of metabolic equivalents of task (METs) for each activity level. To calculate the weekly physical activity (MET-min/week), the number of total minutes dedicated to each activity class was multiplied by the specific MET score for that activity, with 3.3 METs for walking, 4 METs for moderate intensity physical activity, and 8 METs for vigorous intensity physical activity. |
| Smoking status | Current non-smoking. | Smoking status will be assessed by a 32-item questionnaire about smoking, which was adapted from previous research [4]. This questionnaire covers questions such as frequency of smoking, number (current and past) of cigarettes in the past one month, age started smoking, etc. Participants’ smoking status will be categorized as current smoker, former smoker, and never smoking. Nicotine dependence will be assessed using the 6-item Mandarin Chinese version of the Fagerström Test for Nicotine Dependence (FTND) which has been documented to be a useful instrument for evaluating nicotine dependence in Chinese adults [7, 8]. |
| Medication adherence | Self-reported adherence to secondary preventive medications: is defined as adherence to medications that includes dual antiplatelet therapy, beta-blocker, angiotensin converting enzyme inhibitors or angiotensin receptor blockers, and statins [2]. | Medication adherence will be measured by a 8-item Morisky Medication Adherence Scale (MMAS-8) [9]. MMAS-8 is one of the most widely used reliable and valid measures of self-reported medication adherence. The validated Chinese version of the MMAS-8 (C-MMAS-8) has a good internal consistency (Cronbach’s α = 0.77) and test-retest reliability (r = 0.88) [10]. Response choices are yes or no for items 1-7 and a 5-point Likert scale for item 8. Items 1-7 will be scored as “0” or “1”, and item 8 will be scored as “0.2”, “0.4”, “0.6”, “0.8”, or “1”. Scores from the C-MMAS-8 ranged from 0 to 8, the higher the total scores, the better the medication adherence. Prescribed medications will be uploaded to the iCare patients’ App before patients are discharged. Therefore, for patients in the intervention group and control group 1, the medication adherence will be also calculated by iCare patients App. |
| Cardiovascular health | The American Heart Association defines the ideal cardiovascular health as simultaneous presence of four ideal health behaviours and three ideal health factors [11]. The four-ideal health behaviours include non-smoking, body mass index (BMI) < 25 kg/m^2^, physical activity at targeted level, and diet habit being consistent with current guideline recommendations. The three ideal health factors are identified as clinical parameters with untreated blood pressure <120/80 mm Hg, untreated total cholesterol < 5.17 mmol/L (200 mg/dL) and untreated fasting glucose < 5.60 mmol/L (100 mg/dL). | Each component of the cardiovascular health metrics will be dichotomized as 1 (ideal status) or 0 (intermediate or poor status), and the cumulative number of ideal cardiovascular health metrics will be calculated to reflect patients’ overall cardiovascular health (ranged from 0 to 7 points) [11] |
| Anthropometry parameters | Anthropometric parameters include weight (kg), body mass index (kg/m^2^), waist circumference (cm), waist-hip ratio. | All the parameters will be assessed by University affiliated hospitals during follow up. Following standardized procedures, trained study researchers measure the weight, height, waist circumference, hip circumference of all participants using calibrated equipment (SECA 880 scales and SECA 206 wall-mounted metal tapes, SECA North America, Chino, California). Body mass index (BMI) is calculated as weight (kg) divided by height squared (m^2^) [12]. Waist-hip ratio is calculated as waist circumference (cm) divided by hip circumference (cm). |
| Biochemical parameters | Biochemical parameters include total cholesterol, low-density lipoprotein cholesterol, high-density lipoprotein cholesterol, fasting blood glucose, HbA1c, systolic blood pressure, diastolic blood pressure, heart rate. | All the parameters will be assessed by University affiliated hospitals during follow up.  Lipid profile, fasting blood glucose, and HbA1c levels are derived from the laboratory test centers of the study hospitals. Lipid profile and fasting blood glucose are measured with the polyethylene glycol–modified enzyme method, and the glucose oxidase phenol 4-amino antipyrine peroxidase (GOD PAP) method respectively [12].  Blood pressure (BP): Each participant’s seated BP will be measured on the right arm using standard electronic sphygmomanometers by experienced study researchers who attended a 7-day data-collection training session. Three measurements will be obtained with a 30-s interval between cuff inflations if the first measure is normal. Otherwise, participants will be requested to rest for 10 to 30 min before a second measurement is taken. The mean value of 3 measurements is used [12]. |
| 10-year Framingham risk scores (FRS) of cardiovascular risks | The 10-year FRS scores of cardiovascular risks is a model that predicts risk of developing CVD for percipients between 30 to 74 years of age. This model is calculated with input of relevant risk factors such as blood pressure, total cholesterol, HDL cholesterol, smoking status, diabetes, etc. | The 10-year FRS scores of cardiovascular risks will be assessed using the well-validated Framingham risk equation [13]. |
| All cause readmission | Re-admission rate refers to the prevalence of re-admission for all causes during follow-up. | The number of re-admissions will be collected through follow-up visit through reviewing medical reports by researchers, or through self-reporting in the iCare patient App. |
| All-cause mortality | All-cause mortality refers to death due to any and all causes. | The cause of death and the time of death will be obtained by reviewing relevant medical records. |
| Frequency of medical treatment due to CVD | Frequency of medical treatment due to CVD refers to the times of treatment in the hospital for CVD. Doctor-appointed visits and prescriptions are not included. | Frequency of medical treatment due to CVD will be collected through face-to-face clinic follow-up visit through reviewing medical reports by study researchers, or through self-reporting in iCare App. |
| Patients’ engagement in the intervention | Patients’ engagement in the intervention is composed of (1) the length of time participants interacts with the app, (2) the number of screens they visit within the app, (3) the frequency of uploading data in the app, and (4) the number of links they click on while using the app. | The frequency of app use more once a week and the duration of app use more than six months refers to high adherence to app. The frequency of app use more than once a month and the duration of app use more than three months refers to moderate adherence to app. The frequency of app use less than once a month and the duration of app use less than three months refers to low adherence to app. |
| Risk perception | Risk perception is the subjective self-judgement that patients make regarding the probability of occurrence of dangerous events and the severity of a risk. | Risk perception will be assessed using the 4-item version of risk perception questionnaire adapted from Renner & Schwarzer [14]. Items are recorded on a 10-point scale. For example, “How likely is it you will have a cardiac arrest sometime in your life...” |
| Outcome expectation | Outcome expectation are the balance between the pros and cons of certain health behavioral outcomes. | Outcome Expectation of Healthy Diet Scale, Outcome Expectation of Regular Exercise Scale, and Outcome Expectation of Quitting Smoking were from Renner & Schwarzer [14]. Outcome Expectation of Medication Management Scale was developed for this study. |
| Action planning | Action planning describes the time, place and how to implement the intention into action. | Action Planning of Healthy Diet Scale, Action Planning of Regular Exercise Scale, and Action Planning of Quitting Smoking Scale were from Renner & Schwarzer [14]. Action Planning of Medication Management Scale was developed for this study. |
| Coping planning | Coping planning pertains to the anticipation of barriers that might arise in the process of the adoption and maintenance of a behavior, and the degree to which the individual has developed appropriate strategies to cope with such barriers. | Coping Planning of Healthy Diet Scale, Coping Planning of Regular Exercise Scale, and Coping Planning of Quitting Smoking were from Renner & Schwarzer [14]. Coping Planning of Medication Management Scale was developed for this study. |
| Behavioral enjoyment | Behavioral enjoyment refers to one’s comprehensive judgment and emotional response regarding their pleasure, feasibility, and cost for changing a behavior (including the costs of adoption of a new behavior, and the sacrifices to change the original behavior). | Enjoyment of physical activity will be assessed using the 8-item Physical Activity Enjoyment Scale [15]. Enjoyment of healthy eating, smoking cessation, and adherence to medication therapy will be assessed with the a scale adapted from Physical Activity Enjoyment Scale [16]. |
| Social support | Social support is the perception and actuality that one is cared for, has assistance available from other people (family, friends, workers) and that one is part of a supportive social network. | Social support will be assessed using the validated Chinese multidimensional scale of perceived social support (MSPSS) [17], which is comprised of 12-item and three dimensions (supports from friends, family, and significant other). Items will be recorded on a 5-point Likert scale. |
| Self-efficacy | Self-efficacy refers to one’s confidence in being capable of performing a difficult or novel behavior (Action Self-efficacy), keeping up a difficult behavior (Maintenance Self-efficacy or Coping Self-efficacy), or resuming a difficult behavior after an interruption (Recovery Self-efficacy). Action Self-efficacy focuses on initiating such a behavior or performing it once in a lifetime. Maintenance Self-efficacy, a focus on coping with imminent barriers. Recovery Self-efficacy focuses on lapses and regaining confidence after a relapse. | Self-efficacy of diet, physical activity, and smoking cessation will be assessed by scales conducted by Renner & Schwarzer [14]. Self-efficacy of medication therapy adherence will be assessed by a scale adapted from physical activity of Self-efficacy scale. Items are recorded on a 10-point scale. |
| Effectiveness perception | Effectiveness perception refers to one’s perception and experience for the improvement of the health indicators after changing health behaviors. | No existing measure of efficacy perception was available. The efficacy perception will be measured through a 5-item Visual Analogue Scale (ranging from 0 to 10) of effectiveness perception of changes in blood pressure, lipid level, blood glucose, weight, and vascular plaque, which was developed for this study. |
| Intentions | Intentions are personal goals or explicit decisions to act in a certain way, and they focus on a person’s motivation towards a goal in terms of direction and intensity. | Intentions will be assessed using the 4-item Visual Analogue Scale (ranging from 0 to 10) of intention to change unhealthy diet, physical inactivity, smoking, and medication nonadherence adapted from Renner & Schwarzer [14]. |
| Motivation | Motivations to change are direction for behavior, or what causes a person to want to change a behavior. | The motivation will be measured through a 4-item Visual Analogue Scale (ranging from 0 to 10) of motivation to change unhealthy diet, physical inactivity, smoking, and medication nonadherence, which was developed for this study according to Visual Analog Scale for motivation to quit from Vitor de Souza Brangioni et al. [18] |
| Volition | Volitions refer to one’s psychological process of realizing purposes by consciously adjusting their actions. | It will be assessed using a 4-item Visual Analogue Scale (ranging from 0 to 10) of volition to maintain healthy behaviours and medication adherence, which was developed for this study. |
| Stages of behavior change | According to the CAM model, stages of behavior change include precontemplation, contemplation, action, and maintenance stages. | Stages of behavior change are assessed by five-item measures developed by Marcus et al. [19]. For example, the response that “I currently do not exercise, and I do not intend to start exercising in the next 6 months” will be recorded as precontemplation stage; “I currently do not exercise, but I am thinking about starting exercising in the next 6 months” will be recorded as contemplation stage; “I currently exercise some, but not regularly” will be recorded as preparation stage; “I currently exercise regularly, but I have only begun doing so within the last 6 months” will be recorded as action stage; and “I currently exercise regularly, and have done so for longer than 6 months” will be recorded as maintenance stage. Patients at either preparation stage or action stage are regarded as in the action stage in this study. |
| Knowledge of coronary artery disease | Knowledge of coronary artery disease includes knowledge about medical condition, major cardiovascular risk factors, exercise, diet, and psychosocial index. | Patients’ knowledge about coronary artery disease will be assessed using the coronary artery disease education questionnaire (CADE-QII) [20] which is comprised of five dimensions, including medical condition (7-item), risk factors (5-item), exercise (7-item), diet (7-item), and psychosocial index (5-item). |
| Perceived importance of health behaviors | Perceived importance of health behaviors refers to perceptions about the importance of health behaviors. | Participants will be provided with a brief definition of healthy behaviors, then their perceived importance of health behaviors will be measured through a visual analogue scale (VAS) asking participants to rate importance from 0 (not at all important) to 10 (extremely important) [21]. |

**References**

1. Wang Y, Li J, Zheng X, et al. Risk Factors Associated With Major Cardiovascular Events 1 Year After Acute Myocardial Infarction. JAMA Netw Open 2018;1(4):e181079. https://doi.org/10.1001/jamanetworkopen.2018.1079.
2. Piepoli MF, Hoes AW, Agewall S, et al. 2016 European Guidelines on cardiovascular disease prevention in clinical practice: The Sixth Joint Task Force of the European Society of Cardiology and Other Societies on Cardiovascular Disease Prevention in Clinical Practice (constituted by representatives of 10 societies and by invited experts)Developed with the special contribution of the European Association for Cardiovascular Prevention & Rehabilitation (EACPR). Eur Heart J 2016;37(29):2315-2381. https://doi.org/10.1093/eurheartj/ehw106.
3. Chinsese Society of Cardiology. Chinese Expert Consensus on Coronary Heart Disease Rehabilitation/Secondary Prevention. Chin J cardiol 2014(41):1-10.
4. Lv J, Yu C, Guo Y, et al. Adherence to Healthy Lifestyle and Cardiovascular Diseases in the Chinese Population. J Am Coll Cardiol 2017;69(9):1116-1125. https://doi.org/10.1016/j.jacc.2016.11.076.
5. Rubinstein A, Miranda JJ, Beratarrechea A, et al. Effectiveness of an mHealth intervention to improve the cardiometabolic profile of people with prehypertension in low-resource urban settings in Latin America: a randomised controlled trial. Lancet Diabetes Endo 2016;4(1):52-63. https://doi.org/10.1016/s2213-8587(15)00381-2.
6. Ren YJ, Su M, Liu QM, et al. Validation of the Simplified Chinese-character Version of the International Physical Activity Questionnaire-Long Form in Urban Community-dwelling Adults: a Cross-sectional Study in Hangzhou, China. Biomed Environ Sci 2017;30(4):255-263. https://doi.org/10.3967/bes2017.035.
7. Heatherton TF, Kozlowski LT, Frecker RC, Fagerström KO. The Fagerström Test for Nicotine Dependence: a revision of the Fagerström Tolerance Questionnaire. Br J Addict 1991;86(9):1119-1127. https://doi.org/10.1111/j.1360-0443.1991.tb01879.x.
8. Yang, Shiffman S, Rockett IRH, Cui X, Cao R. Nicotine Dependence among Chinese City Dwellers: A Population-Based Cross-Sectional Study. Nicotine Tob Res 2011;13(7):556-564. https://doi.org/10.1093/ntr/ntr040.
9. Morisky DE, Green LW, Levine DM. Concurrent and predictive validity of a self-reported measure of medication adherence. Med Care 1986;24(1):67-74. https://doi.org/10.1097/00005650-198601000-00007.
10. Yan J, You LM, Yang Q, et al. Translation and validation of a Chinese version of the 8-item Morisky medication adherence scale in myocardial infarction patients. J Eval Clin Pract 2014;20(4):311-317. https://doi.org/10.1111/jep.12125.
11. Lloyd-Jones Donald M, Hong Y, Labarthe D, et al. Defining and Setting National Goals for Cardiovascular Health Promotion and Disease Reduction. Circulation 2010;121(4):586-613. https://doi.org/10.1161/CIRCULATIONAHA.109.192703.
12. Li Y, Wang DD, Ley SH, et al. Potential Impact of Time Trend of Life-Style Factors on Cardiovascular Disease Burden in China. J Am Coll Cardiol 2016;68(8):818-833. https://doi.org/10.1016/j.jacc.2016.06.011.
13. D'Agostino RB, Sr., Vasan RS, Pencina MJ, et al. General cardiovascular risk profile for use in primary care: the Framingham Heart Study. Circulation 2008;117(6):743-753. https://doi.org/10.1161/circulationaha.107.699579.
14. Schwarzer BRR. Risk and Health Behaviors——Documentation of the Scales of the Research Project: Risk Appraisal Consequences in Korea(RACK). (2ed Edition). 2005. http://www.gesundheitsrisiko.de/docs/RACKEnglish.pdf.
15. Kendzierski D, Decarlo KJ. Physical Activity Enjoyment Scale: Two validation studies. J Sport Exercise Psy 1991;13(1):50-64.
16. Mcauley E, Duncan T, Tammen VV. Psychometric properties of the Intrinsic Motivation Inventory in a competitive sport setting: a confirmatory factor analysis. Res Q Exercise Sport 1989;60(1):48.
17. Zhou K, Li H, Wei X, et al. Reliability and validity of the multidimensional scale of perceived social support in Chinese mainland patients with methadone maintenance treatment. Compr Psychiat 2015;60:182-188. https://doi.org/10.1016/j.comppsych.2015.03.007.
18. Vitor de Souza Brangioni MC, Pereira DA, Thibaut A, Fregni F, Brasil-Neto JP, Boechat-Barros R. Effects of Prefrontal Transcranial Direct Current Stimulation and Motivation to Quit in Tobacco Smokers: A Randomized, Sham Controlled, Double-Blind Trial. Frontiers in Pharmacology. 2018; 9(14).
19. Marcus BH, Selby VC, Niaura RS, Rossi JS. Self-efficacy and the stages of exercise behavior change. Res Q Exerc Sport 1992;63(1):60-66. https://doi.org/10.1080/02701367.1992.10607557.
20. Ghisi GL, Grace SL, Thomas S, Evans MF, Oh P. Development and psychometric validation of the second version of the Coronary Artery Disease Education Questionnaire (CADE-Q II). Patient Educ Couns 2015;98(3):378-383. https://doi.org/10.1016/j.pec.2014.11.019.
21. Gillison F, Stathi A, Reddy P, et al. Processes of behavior change and weight loss in a theory-based weight loss intervention program: a test of the process model for lifestyle behavior change. Int J Behav Nutr Phy 2015;12:2. https://doi.org/10.1186/s12966-014-0160-6.
