# Supplementary material for: Individualized mobile health interventions for cardiovascular event prevention in patients with coronary heart disease: study protocol for the iCARE randomized controlled trial
Source: BMC Cardiovasc Disord. 2021 Jul 13;21:340. doi: 10.1186/s12872-021-02153-9 (PMC8278759; doi:10.1186/s12872-021-02153-9)
Supplement: Supplementary file 3 — Additional file 3. Questionnaires for the iCARE trial. [file 12872_2021_2153_MOESM3_ESM.pdf]

QUESTIONNAIRES FOR THE ICARE TRIAL

(2021/6/10)

Individualized Mobile Health Interventions for Cardiovascular Event Prevention in Patients with Coronary Heart Disease: Study Protocol for the iCARE Randomized Controlled Trial

Contents

Baseline Assessment-A......2
Baseline Assessment-B......8
Short Food Frequency Questionnaire (FFQ)......13
International Physical Activity Questionnaire......17
Smoking Status Questionnaire......20
Eight-item Morisky Medication Adherence Scale (MMAS-8)......26
Stages of Behavior Change......27
Follow-up Assessment Questionnaire......28
Mediators and Moderators Questionnaire......31
Understanding of the Importance of Healthy Lifestyles......57
Coronary Artery Disease Education Questionnaire (CADE−Q)......59

Baseline Assessment-A

A1. Nickname: ________________________________

A2. Gender: ○ 1. Male ○ 2. Female

A3. Age: ______

A4. Nationality: ○ 1. Han ○ 2. Hui ○ 3. Tibetan ○ 4. Uygur ○ 5. Dai ○ 6. Others ______

A5. Marriage: ○ 1. Married ○ 2. Unmarried ○ 3. Divorced / separated ○ 4. Widowed

A6. Who live with:

□ 1. Living alone (mutually exclusive option) □ 2. Spouse / partner □ 3. Children □ 4. Parents □ 5. Brothers and sisters □ 6. Relatives □ 7. Babysitter □ 8. Other _______

A7. Who is your closest person? _____

A7.1 What is the relationship between this person and you?

○1. Spouse / partner

○2. Children

○3. Parents

○4. Brothers and sisters

○5. Relatives

○6. Babysitter

○ 7. Friend

○ 8. Other _________

A8. Education level:

○1. Elementary school and below

○2. Junior high school

○ 3. High school

○4. Junior college

○5. Bachelor degree and above

A9. Your current occupation:

○ 1. Retired (retired) personnel ○ 2. National civil servants ○ 3. Professional and technical personnel ○ 4. Staff ○ 5. Enterprise management personnel ○ 6. Workers ○ 7. Farmers ○ 8. Students ○ 9. Active-duty military ○ 10. Freelancers ○ 11. Self-employed ○ 12. Unemployed ○ 13. Medical staff ○ 14. The professionals (such as accountants, architects, reporters, etc.) ○ 15. Others ________________

A10. Family income (specific values): ____ RMB/month

A10.1 Monthly household income?

○1. Less than 1800 RMB/month

○2.1800-3000 RMB/month

○3.3000-8000 RMB/month

○4. More than 8,000 RMB/month

A11. Medical insurance payment:

□1. Public expense

□ 2. Medical insurance

□3. New Rural Cooperative

□4. At your own expense

□5. Commercial Insurance

□6. One old and one young

□ 7. Other ________________

A12. Height:_______ cm

A13. Weight:_______ kg

A13.1 Body Mass Index (BMI): ______kg/m^2

A14. Waist circumferences: _______ cm

A15. Hip circumferences: _______ cm

A15.1 Waist-to-hip ratio: ________

A16. Past history:

A16a. Diabetes:

○1. No (please skip to question A16b)

○ 2. Yes

A16a1. Types of diabetes:

○1. Type I

○ 2. Type II

A16a2. Diabetes history: ______ years ______ months

A16a3. Whether to take medication or insulin treatment before hospitalization:

○1. No

○2. Yes

A16b. Hypertension:

○1. No (please skip to question A16c)

○ 2. Yes

A16b1. Hypertension classification:

○1. Normal blood pressure (systolic blood pressure < 120mmHg and diastolic blood pressure < 80mmhg)

○2. High normal value (systolic blood pressure 120~139mmHg and / or diastolic blood pressure 80~89mmHg)

○3. Hypertension (systolic blood pressure ≥ 140mmHg and / or diastolic blood pressure ≥ 90mmHg)

A16b2. Risk stratification of hypertension:

○ 1. Low risk ○ 2. Medium risk ○ 3. High risk ○ 4. Very high risk ○ 5. None

A16b3. History of hypertension: ______ years ______ months

A16b4. Does the patient take antihypertensive medication before hospitalization?

○1. No

○2. Yes

A16c. Hyperlipidemia:

○1. No (please skip to question A16d)

○ 2. Yes

A16c1. History of hyperlipidemia: ______ years ______ months

16c2. Does the patient take lipid medication before hospitalization?

○1. No

○2. Yes

A16d. Cerebrovascular disease:

○1. No (please skip to question A16e)

○ 2. Yes

A16d1. Cerebrovascular disease history: ______ years ______ months

A16d2. Does the patient take medication before hospitalization?

○1. No

○2. Yes

A16e. Do you have the following past history?

□ 1. None □ 2. Gastric ulcer □ 3. Glaucoma □ 4. Cancer

□ 5.COPD □ 6. Other: _________________

A17. Family history:

□ 1. None □ 2. Coronary heart disease □ 3. Diabetes □ 4. Hypertension

□ 5. Hyperlipidemia □ 6. Cerebrovascular disease □ 7. Others: _________________

A18. Does the patient need help in indoor activities?

○1. Can walk independently on flat ground for 45 meters

○2. Need some help

○3. Need a lot of help

○4. Totally dependent on others

A19. Does the patient need help going up and down the stairs?

○1. Independent up and down stairs (10-15 steps up and down)

○2. Need some help (need to hold the stairs, others to support, or use crutches, etc.)

○3. Need a lot of help or rely entirely on others

A20. Does the patient use the following equipment or appliances?

□ 1. None □ 2. Crutches □ 3. Walker

□ 4. Prosthesis □ 5. The wheelchair or scooter □ 6. Others _________________

A21. Current residential address: ________________________________

A22. Contact number (patient): ________________________________

A23. Contact number (family): ________________________________

Baseline Assessment-B

B1. History of heart disease and heart surgery (fill in before surgery)

B1a. Atrial fibrillation or atrial flutter:

○1. No

○2. Yes

B1b. Myocardial infarction:

○1. No

○2. Yes

B1c. Angina:

○1. No

○2. Yes

B1d. Ventricular arrhythmia:

○1. No

○2. Yes

B1e. Atrioventricular block:

○ 1. No
○ 2. Yes

B1f. Heart failure:

○1. No

○2. Yes

B1g. Was coronary heart disease diagnosed before this hospitalization?

○ 1. No (skip to B1h)

○ 2. Yes

B1g1. History of coronary heart disease: ________ years ________ months

B1h. History of cardiac surgery:

○1. No (please skip to question B1j)

○2. Yes

B1i. Which of the following heart surgeries have you had before this hospital admission?

□1. Percutaneous coronary stent implantation

□2. Heart bypass surgery (Coronary Artery Bypass Grafting)

□3. Heart valve surgery

□4. Pacemaker implantation

□5. Heart transplantation

□6. Other

B1j. Aortic reflux:

○1. No

○2. Yes

B1k. Mitral regurgitation:

○1. No

○2. Yes

B1l. Tricuspid regurgitation:

○1. No

○2. Yes

B1m. Pulmonary valve regurgitation:

○1. No

○2. Yes

B1n. Pulmonary hypertension:

○1. No

○2. Yes

B1o. Left ventricular ejection fraction (LVEF): __________%

B2. Baseline assessment - postoperative cardiac factors related data

B2. Diagnosis and treatment of this admission (fill in after operation)

B2a. Diagnosis type:

○ 1. Stable angina pectoris

○ 2. Unstable angina pectoris

○ 3. Acute ST- segment elevation myocardial infarction

○ 4. Acute ST- segment elevation myocardial infarction

○ 5. Acute Coronary Syndrome (ACS)

○ 6. Asymptomatic myocardial ischemia

○ 7. Other _________________

B2b1. Killip classification:

○ 1. I

○ 2. II

○ 3. III

○ 4. IV

B2b2. CCS classification:

○ 1. I

○ 2. II

○ 3. III

○ 4. IV

B2b3. NYHA classification:

○ 1. I

○ 2. II

○ 3. III

○ 4. IV

B2c. Results of Coronary Angiography (CAG): ____

1. Left anterior descending artery disease (LAD):________ %

2. Circumflex branch disease (LCX):________%
3. The left main coronary artery (LM):________%
4. Right coronary artery disease (RCA):________%
The left coronary artery disease (LCA):________%
6. Blunt marginal branch disease (OM):________ %
7. Diagonal branch disease (D): ________ %
8. Spacer (S): ________ %
9. Posterior descending branch (PDA):_________%
10. Posterior branch of left ventricle (PLA):___________%

B2d. Treatment received:

B2d1. Percutaneous Coronary Intervention (PCI):

○1. No (please skip to question B2d2)

○2. Yes

B2d1a. Number of implanted stents: ______ pieces

B2d1b. Vessel site for stent placement:

□ 1. Left anterior descending branch (LAD)
□ 2. Circumflex branch (LCX)
□ 3. Left main trunk (LM)
□ 4. Right coronary artery (RCA)
□ 5. Left coronary artery (LCA)
□ 6. Obtuse marginal branch (OM)
□ 7. Diagonal branch (D)
□ 8. Septal branch (S)
□ 9. Posterior descending branch (PDA)
□ 10. Posterior branch of left ventricle (PLA)

B2d1c. PCI operation date: __________Year/Month/Day

Short Food Frequency Questionnaire (FFQ)

In the past week, what was the average daily amount of the following types of foods you consumed?

D 1.1.1 How often do you eat staple food?

○1. Every day

○2. 4-6 days a week

○3. 1-3 days a week

○4. Several times per month

○5. Don't eat or eat very little

D 1.1.2 How many staple foods do you eat on average every day?

○1. Less than 250g

○2. 250g-400g

○3. Greater than 400g

D 1.2.1 How often do you eat vegetables?

○1. Every day

○2. 4-6 days a week

○3. 1-3 days a week

○4. Several times per month

○5. Don't eat or eat very little

D1.2.2 How many vegetables do you eat on average every day?

○1. Less than 300g (less than half a plate of vegetables)

○2. 300g-500g (half plate to 1 plate of vegetables)

○3. More than 500g (more than 1 plate of vegetables)

D 1.3.1 How often do you eat fruits?

○1. Every day

○2. 4-6 days a week

○3. 1-3 days a week

○4. Several times per month

○5. Don't eat or eat very little

D 1.4.1 How often do you eat fish and shrimp?

○1. Every day

○2. 4-6 days a week

○3. 1-3 days a week

○4. Several times per month

○5. Don't eat or eat very little

D1.4.2 How much fish and shrimp do you eat on average every day?

○1. Less than 100g

○2. 100g-200g

○3. More than 200g

D 1.5.1 How often do you eat poultry and meat?

○1. Every day

○2. 4-6 days a week

○3. 1-3 days a week

○4. Several times per month

○5. Don't eat or eat very little

D1.5.2 How much fish and shrimp do you eat on average every day?

○1. Less than 100g

○2. 100-200g

○ 3. More than 200g

D 1.6.1 How often do you eat eggs and duck eggs?

○1. Every day

○2. 4-6 days a week

○3. 1-3 days a week

○4. Several times per month

○5. Don't eat or eat very little

D1.6.2 How many eggs or duck eggs do you eat on average every day?

○1. Less than half an egg

○2. 1 egg

○3. More than 1 egg

D 1.7.1 What is your taste for edible salt?

○1. Very salty

○2. A little salty

○3. Moderate

○4. A little light

○5. Very light

D 1.8.1 For cooking oil, your tastes are?

○1. Very oily

○2. A little oily

○3. Moderate

○4. A little light

○5. Very light

International Physical Activity Questionnaire

We are interested in finding out about the kinds of physical activities that people do as part of their everyday lives. The questions will ask you about the time you spent being physically active in the **last 7 days**. Please answer each question even if you do not consider yourself to be an active person. Please think about the activities you do at work, as part of your house and yard work, to get from place to place, and in your spare time for recreation, exercise or sport.

Think about all the **vigorous** activities that you did in the **last 7 days**. **Vigorous** physical activities refer to activities that take hard physical effort and make you breathe much harder than normal. Think *only* about those physical activities that you did for at least 10 minutes at a time.

1. During the **last 7 days**, on how many days did you do **vigorous** physical activities like heavy lifting, digging, aerobics, or fast bicycling?

   _____ **days per week**

   ☐ vigorous physical activities ***Skip to question 3***

2. How much time did you usually spend doing **vigorous** physical activities on one of those days?

   _____ **hours per day**

   _____ **minutes per day**

   ☐ Don't know/Not sure

Think about all the **moderate** activities that you did in the **last 7 days**. **Moderate** activities refer to activities that take moderate physical effort and make you breathe somewhat harder than normal. Think only about those physical activities that you did for at least 10 minutes at a time.

3. During the **last 7 days**, on how many days did you do **moderate** physical activities like carrying light loads, bicycling at a regular pace, or doubles tennis? Do not include walking.

_____ **days per week**

☐ moderate physical activities ***Skip to question 5***

4. How much time did you usually spend doing **moderate** physical activities on one of those days?

_____ **hours per day**

_____ **minutes per day**

☐ Don't know/Not sure

Think about the time you spent **walking** in the **last 7 days**. This includes at work and at home, walking to travel from place to place, and any other walking that you have done solely for recreation, sport, exercise, or leisure.

5. During the **last 7 days**, on how many days did you **walk** for at least 10 minutes at a time?

_____ **days per week**

☐ walking ***Skip to question 7***

6. How much time did you usually spend **walking** on one of those days?

_____ **hours per day**

_____ **minutes per day**

☐ Don't know/Not sure

The last question is about the time you spent **sitting** on weekdays during the **last 7 days**. Include time spent at work, at home, while doing course work and during leisure time. This may include time spent sitting at a desk, visiting friends, reading, or sitting or lying down to watch television.

7. During the **last 7 days**, how much time did you spend **sitting** on a **week day**?

_____ **hours per day**

_____ **minutes per day** ☐ Don’t know/Not sure

Smoking Status Questionnaire

D3a. Regarding smoking, which of the following conditions is more suitable for you?

○ 1. Smoke every day (please skip to question D3b)

○ 2. Suck occasionally, not every day (please skip to question D3b)

○ 3. Previously smoked, but have quit smoking (please skip to question D3 a.1)

○ 4. Never smoke (please skip to question D4a)

D3a.1 How long do you quit smoking: _______ year ______ month ______ day

D3b. How long have you been smoking: _______ year ______ month

D3c. How many cigarettes do your smoke every day? _________

D3f. Under what circumstances do you usually smoke? [Multiple choice] *

□ 1. At home □ 2. At work □ 3. At leisure □ 4. When you are boring

□ 5. When you want to concentrate □ 6. When you feel nervous

□ 7. When you are lonely □ 8. When you are with other people who smoke

□ 9. After meals □ 10. When drinking alcohol □ 11. When driving

□ 12. When talking on the phone □ 13. When you feel stressed

□ 14. When you feel anxious □ 15. Others _________________*

D3p. Duration to smoke a cigarette: _______ minutes

D3g1. How soon after you wake up do you smoke your first cigarette?

○ 1. Within 5 minutes ○ 2. 6 to 30 minutes ○ 3. 31 to 60 minutes ○ 4. After 60 minutes

D3g2. Do you find it difficult to refrain from smoking in places where it is forbidden (e.g., in church, at the library, in the cinema)?

○ 1. Yes ○ 2. No

D3g3. Which cigarette would you hate most to give up?

○ 1. The first one in the morning ○ 2. Any Other

D3g4. How many cigarettes per day do you smoke?

○ 1. ≤ 10 branched ○ 2.11-20 branched ○ 3.21-30 branched ○ 4. 31 or more

D3g5. Do you smoke more frequently during the first hours after waking than during the rest of the day?

○ 1. Yes ○ 2. No

D3g6. Do you smoke when you are so ill that you are in bed most of the day?

○ 1. Yes ○ 2. No

D3h1. Have you tried to quit smoking?

○1. No (please skip to question D3i)

○2. Yes

D3h2. Have you tried many times? _______

D3h3. When was the last time you seriously quit smoking?

○ 1. Within the last 30 days ○ 2. 1-6 months ago

○ 3.7-12 months ago ○ 4. More than 1 year ago

D3h4. The last time you quit smoking, how long did you insist on not smoking?

○ 1. Less than 1 day ○ 2.1-30 days ○ 3. More than 1 month, less than 6 months

○ 4.6-12 months ○ 5.12 months or more

D3h5. What method did you use to quit smoking?

□ 1. Ask medical staff for help □ 2. Use self-help smoking cessation materials

□ 3. Use smoking cessation drugs, specifically ________*

□ 4. With perseverance □ 5. Others, please specify _________________*

D3h6. Have you ever used smoking cessation products to help you quit smoking?

○1. No (please skip to question D3h8)

○ 2. Yes

D3h7. Which smoking cessation product have you used?

□ 1. Nicotine gum □ 2. Nicotine patch □ 3. Bupropion hydrochloride

□ 4. Varenicline tartrate □ 5. Chinese herbal medicine / Chinese medicine acupuncture

□ 6. Electronic cigarette □ 7. Other _________________*

D3h8. Do you relapse after quitting smoking?

○1. No (please skip to question D3i)

○2. Yes

D3h9. You Relapse reasons?

□ 1. The onset of addiction is difficult to overcome

□ 2. The influence of other smokers around

□ 3. My mood is particularly negative and I have no confidence in quitting smoking

□ 4. Work pressure

□ 5. Tension, anxiety, depression

□ 6. Weight gain after quitting smoking

□ 7. High interpersonal relationship or social pressure

□ 8. Others, please specify ________________*

D3i. Is there a smoker among the people you are in frequent contact with?

○1. No (please skip to question D3i.2)

○2. Yes

D3i.1 If yes, who are smokers

□1. Co-resident

□2. Partner

□3. Friends

□4. Colleagues

D3i.2 Is smoking allowed in your work environment?

○1. No

○2. Yes

D3j1. Do you quit smoking during hospitalization?

○1. No (please skip to question D3k1)

○2. Yes

D3k1. If you quit smoking, how difficult is it for you to quit? (0 means the least difficulty, 10 means the most difficult) ______________________*

D3k2. How important is successful smoking cessation to you? (0 means the least importance, 10 means the most importance) ______________________*

D3k3. What will you do after you leave the hospital?

○1. Tobacco control (reduce smoking)

○2. Quit smoking (skip to D3k5)

○3. Continue to maintain current smoking status (skip to D3k7)

D3k4. If you control tobacco, how many cigarettes do you want to reduce per week? ____________

D3k5. Why do you want to quit smoking / control smoking?

□ 1. Reduce the harm caused by smoking to my body

□ 2. Reduce the possibility of re-hospitalization

□ 3. To make yourself look more energetic

□ 4. The doctor and nurse told me to quit smoking / control smoking

□ 5. My family wants me to quit smoking / control smoking

□ 6. Others, please specify ___________________

D3k6. How confident are you in quitting /controlling smoking this time? (0 means the least confidence, 10 means the most confidence________ *

D3k7. Why do you not want to quit smoking?

□ 1. Smoking is not harmful to my body

□ 2. There is no benefit in quitting smoking

□ 3. Have tried to quit smoking but the onset of addiction is difficult to overcome

□ 4. The influence of other smokers around

□ 5. I have no confidence in quitting smoking, and I am afraid of failing to quit smoking

□ 6. The work pressure is high, and smoking needs to be used to relieve the pressure

□ 7. Have significantly increased weight after quitting smoking

□ 8. High interpersonal relationship or social pressure

□ 9. Other _________________*

Eight-item Morisky Medication Adherence Scale (MMAS-8)

1. Do you sometimes forget to take your coronary heart disease pills?
○1. No
○2. Yes
2. Over the past two weeks, were there any days when you did not take your coronary heart disease medicine?
○1. No
○2. Yes
3. Have you ever cut back or stopped taking your medication without telling your doctor because you felt worse when you took it?
○1. No
○2. Yes
4. When you travel or leave home, do you sometimes forget to bring along your medications?
○1. No
○2. Yes
5. Did you take your coronary heart disease medicine yesterday?
○1. No
○2. Yes
6. When you feel like your coronary heart disease is under control, do you sometimes stop taking your medicine?
○1. No
○2. Yes
7. Taking medication everyday is a real inconvenience for some people. Do you ever feel hassled about sticking to your coronary heart disease treatment plan?
○1. No
○2. Yes
8. How often do you have difficulty remembering to take all your coronary heart disease medication?
○1. Very difficult
○2. Difficulty
○3. General
○4. Easy
○5. Very easy

Stages of Behavior Change

F1. For eating habit, please choose the situation that best suits you:
○1. I currently do not eat healthily, and I do not intend to start eat healthily in the next 6 months
○2. I currently do not eat healthily, but I am thinking about starting eat healthily in the next 6 months
○3. I currently eat healthily some, but not regularly
○4. I currently eat healthily regularly, but I have only begun doing so within the last 6 months
○5. I currently eat healthily regularly, and have done so for longer than 6 months

F2. For exercise, please choose the situation that best suits you:
○1. I currently do not exercise, and I do not intend to start exercising in the next 6 months
○2. I currently do not exercise, but I am thinking about starting exercising in the next 6 months
○3. I currently exercise some, but not regularly
○4. I currently exercise regularly, but I have only begun doing so within the last 6 months
○5. I currently exercise regularly, and have done so for longer than 6 months

F3. For eating habit, please choose the situation that best suits you:
○1. I currently do not stop smoking, and I do not intend to start stop smoking in the next 6 months
○2. I currently do not stop smoking, but I am thinking about starting stop smoking in the next 6 months
○3. I currently stop smoking some, but not regularly
○4. I currently stop smoking regularly, but I have only begun doing so within the last 6 months
○5. I currently stop smoking regularly, and have done so for longer than 6 months

F4. For taking medicine, please choose the situation that best suits you:
○1. I currently do not take medicine regularly, and I do not intend to start taking medicine regularly in the next 6 months
○2. I currently do not take medicine regularly, but I am thinking about starting taking medicine regularly in the next 6 months
○3. I currently take medicine some, but not regularly
○4. I currently take medicine regularly, but I have only begun doing so within

the last 6 months
○5. I currently take medicine regularly, and have done so for longer than 6 months

Follow-up Assessment Questionnaire

Basic Information
SF1. Survey method:<br>○1. Face-to-face survey ○2. Telephone follow-up ○3. APP follow-up ○4. WeChat follow-up<br>SF2. Survey Time: _____ year/month/day<br>SF3. Follow-up timepoints:<br>○1. One month ○2. Three months ○3. Six months ○4. Nine months<br>○5. Twelve months ○6. Twenty-four months ○7. Thirty-six months<br>SF4. Did the patient die during the follow-up period?<br>○1. Yes (cause of death: ______________) ○2. No<br>SF5. Did the patient lose to follow-up?<br>○1. Yes (reason for loss to follow-up: ______________) ○2. No<br>Note: In case of loss to follow-up or death, the following investigation shall be terminated.
Vital signs
SF6. Systolic blood pressure: ____mmHg<br>SF7. Diastolic blood pressure: ____mmHg<br>SF8. Heart rate: __________bpm<br>SF9. Weight: ______kg<br>SF10.BMI:______kg/m^2<br>SF11. Hip circumference: _________cm<br>SF12. Waist circumference: _________cm
Physical condition (since last follow-up)
SF13. Angina: ○1. Yes ○2. No<br>SF13.1 If yes, the timing of occurrence:

○ 1. At rest ○ 2. During physical activity ○ 3. The frequency gradually increases<br>SF13.2 Did the patient take nitroglycerin? ○ 1. Yes ○ 2. No
SF14. Shortness of breath: ○ 1. Yes ○ 2. No<br>SF14.1 If yes, ○1. Intermittent ○2. Continuous<br>SF14.2 If yes, the timing of occurrence: ○1. At rest ○2. During physical activity
SF15. Acute myocardial infarction: ○1. Yes ○2. No
SF16. New or worsening heart failure: ○1. Yes ○2. No
SF17. Readmitted: ○1. Yes ○ 2. No<br>SF17.1 If yes, the reason is:<br>○1. Coronary heart disease ○2. Stroke ○3. Peripheral vascular disease<br>○4. Atrial fibrillation ○5. Heart failure<br>○6. Diabetes ○7. Surgery (If yes, continue to answer SF17.2) ○8. Others, ______<br>SF17.2 Type of operation:<br>○1. PCI ○2. CABG ○3. Radiography ○4. Others, ______
SF18. Since the last follow-up, has the patient been in the outpatient or emergency department (except for follow-up visits designated by the doctor)?<br>○1. Yes (continue to answer) ○ 2. No<br>SF18.1 How often do patients go to the outpatient and emergency departments (except for follow-up visits specified by the doctor)? _____ times<br>SF18.2 What is the reason for patients in the outpatient and emergency department (except for follow-up visits designated by the doctor)?<br>○ 1. Cardiac ischemic events ○ 2. Other ________
SF19. Since the last follow-up to the present, the cost of participating in this study: ______ RMB
Laboratory test results
SF20. Laboratory testing: ○1. Yes ○2. No (skip to the next question)<br>SF20.1 Total Cholesterol _______mmol/L<br>SF20.2 Low-density Lipoprotein Cholesterol ______mmol/L

SF20.3 High Density Lipoprotein Cholesterol _______mmol/L<br>SF20.4 Triglyceride __________mmol/L<br>SF20.5 Fasting blood glucose ________ mmol/L<br>SF20.6 Glycated hemoglobin __________%<br>SF20.7 Echocardiography: LVEF _____________%
Other
SF21. Heart age: _______ years old
SF22. Ten-year risk of cardiovascular events: ________%

Mediators and Moderators Questionnaire

BI. Intention

The following questions are about your level of intention to exercise regularly, eat a healthy diet, quit smoking, and regularly take medication prescribed by your doctor. The two ends of the line indicate "no plan at all" (0) and "strong plan (10)" respectively. Which intentions do you have for the next weeks and months?

BI-1. Do you intend to exercise regularly in the next few weeks and months?<br>0 1 2 3 4 5 6 7 8 9 10
BI-2. Do you intend to eat healthy in the next few weeks and months?<br>0 1 2 3 4 5 6 7 8 9 10
BI-3. Do you intend to quit smoking in the next few weeks and months?<br>0 1 2 3 4 5 6 7 8 9 10
BI-4. Do you intend to take medicine of coronary heart disease in the next few weeks and months?<br>0 1 2 3 4 5 6 7 8 9 10
BI-5. The total score of intention is ________

RP. Risk Perception

The following questions are about your perception of your risk of a cardiac event. The two ends of the line indicate "impossible" (0) and "very likely (10)" respectively. The higher the value, the higher the risk you think you are.

RP-1. How likely is it you will have myocardial infarction or reoccurrence after stenting/re-occlusion after thrombolysis sometime in your life?<br>0 1 2 3 4 5 6 7 8 9 10

RP-2. How likely it it you will have sudden death sometime in your life?

0 1 2 3 4 5 6 7 8 9 10

Notes: Sudden cardiac death: a natural death caused by cardiac causes that occurs within 1 hour after the onset of acute symptoms (such as myocardial infarction), characterized by loss of consciousness.

RP-3. How likely is it you will have heart failure sometime in your life?

0 1 2 3 4 5 6 7 8 9 10

Notes: Heart failure: severe breathing difficulties, edema of lower limbs, holding breath after exercise, etc.

RP-4. How likely is it you will have arrhythmia sometime in your life?

0 1 2 3 4 5 6 7 8 9 10

Notes: arrhythmia: irregular pulse, palpitations, etc.

RP-5. The total score of risk perception is: ________

OE. Outcome Expectancies

OE-D. Outcome expectancies of healthy diet

What do you think, what will be the consequences if you change your nutrition to low-fat or low salt food? The two ends of the line indicate "not at all true" (0) and "exactly true (10)" respectively.

OE-D-1. If I eat healthy foods (low-fat or low-salt), I'll feel physically more attractive.

0 1 2 3 4 5 6 7 8 9 10

OE-D-2. If I eat healthy foods (low-fat or low-salt), I won't have weight problems (anymore).

0 1 2 3 4 5 6 7 8 9 10

OE-D-3. If I eat healthy foods (low-fat or low-salt), food won't taste as good.

0 1 2 3 4 5 6 7 8 9 10

OE-D-4. If I eat healthy foods (low-fat or low-salt), that will impair my social life (at parties, with friends).

0 1 2 3 4 5 6 7 8 9 10

OE-D-5. If I eat healthy foods (low fat or low salt), that will be good for my blood pressure.

0 1 2 3 4 5 6 7 8 9 10

OE-D-6. If I eat healthy foods (low-fat or low-salt), I feel more comfortable in my heart.

0 1 2 3 4 5 6 7 8 9 10

OE-D-7. If I eat healthy foods (low-fat or low-salt), I'll have to make an effort of buying the right products.

0 1 2 3 4 5 6 7 8 9 10

OE-D-8. If I eat healthy foods (low-fat or low-salt), that will be good for my cholesterol level.

0 1 2 3 4 5 6 7 8 9 10

OE-D-9. If I eat healthy foods (low-fat or low-salt), I'll have to spend more time on preparing meals.

0 1 2 3 4 5 6 7 8 9 10

OE-D-10. If I eat healthy foods (low-fat or low-salt), that will mean a loss of life quality for me.

0 1 2 3 4 5 6 7 8 9 10

OE-D-11. If I eat healthy foods (low-fat or low-salt), that will be a burden for my financial situation.

0 1 2 3 4 5 6 7 8 9 10

OE-D-12. If I eat healthy foods (low-fat or low-salt), other people will appreciate my willpower.

0 1 2 3 4 5 6 7 8 9 10

OE-D-13. The total score is: ______

OE-E. Exercise Outcome expectancies

What do you think, what will be the consequences if you exercise regularly? The two ends of the line indicate "not at all true" (0) and "exactly true (10)" respectively.

OE-E-1. If I exercise regularly, I will simply feel better afterwards.

0 1 2 3 4 5 6 7 8 9 10

OE-E-2. If I exercise regularly, I won’t have weight problems (anymore).

0 1 2 3 4 5 6 7 8 9 10

OE-E-3. If I exercise regularly, I’ll have to make a great effort each time.

0 1 2 3 4 5 6 7 8 9 10

OE-E-4. If I exercise regularly, other people will appreciate my willpower.

0 1 2 3 4 5 6 7 8 9 10

OE-E-5. If I exercise regularly, my cholesterol level will improve.

0 1 2 3 4 5 6 7 8 9 10

OE-E-6. If I exercise regularly, I’ll look more attractive.

0 1 2 3 4 5 6 7 8 9 10

OE-E-7. If I exercise regularly, I’ll be more balanced in my daily life.

0 1 2 3 4 5 6 7 8 9 10

OE-E-8. If I exercise regularly, that will mean an increase of life quality.

0 1 2 3 4 5 6 7 8 9 10

OE-E-9. If I exercise regularly, I’ll have to take a lot of time each time.

0 1 2 3 4 5 6 7 8 9 10

OE-E-10. If I exercise regularly, I will be appreciated by others for that.

0 1 2 3 4 5 6 7 8 9 10

OE-E-11. If I exercise regularly, that will be a burden for my financial situation.

0 1 2 3 4 5 6 7 8 9 10

OE-E-12. If I exercise regularly, I prevent a heart attack.

0 1 2 3 4 5 6 7 8 9 10

OE-E-13. If I exercise regularly, that will be good for my blood pressure.

0 1 2 3 4 5 6 7 8 9 10

OE-E-14. The total score is: ______

OE-S. Outcome expectancies of quitting smoking

What do you think, which consequences will arise if you do not smoke? The two ends of the line indicate "not at all true" (0) and "exactly true (10)" respectively.

OE-S-1. If I quit smoking, I will be nervous and unbalanced.

0 1 2 3 4 5 6 7 8 9 10

OE-S-2. If I quit smoking, that will be good for my blood pressure.

0 1 2 3 4 5 6 7 8 9 10

OE-S-3. If I quit smoking, that will unburden me financially.

0 1 2 3 4 5 6 7 8 9 10

OE-S-4. If I quit smoking, I might prevent a heart attack.

0 1 2 3 4 5 6 7 8 9 10

OE-S-5. If I quit smoking, I will be more attractive for others (whiter teeth, better skin, nicer smell of clothes).

0 1 2 3 4 5 6 7 8 9 10

OE-S-6. If I quit smoking, I will simply feel better physically.

0 1 2 3 4 5 6 7 8 9 10

OE-S-7. If I quit smoking, that will impair my social life (e.g. at parties, with friends).

0 1 2 3 4 5 6 7 8 9 10

OE-S-8. If I quit smoking, my cholesterol level will improve.

0 1 2 3 4 5 6 7 8 9 10

OE-S-9. If I quit smoking, that will mean a loss of life quality to me.

0 1 2 3 4 5 6 7 8 9 10

OE-S-10. If I quit smoking, I will gain weight.

0 1 2 3 4 5 6 7 8 9 10

OE-S-11. If I quit smoking, others will appreciate my willpower.

0 1 2 3 4 5 6 7 8 9 10

OE-S-12. The total score is: ______

OE-M. Outcome expectancies of taking prescribed medicine for coronary heart disease

What do you think, which consequences will arise if you take prescribed medicine? The two ends of the line indicate "not at all true" (0) and "exactly true (10)" respectively.

OE-M-1. If compliance with prescribed medicine, I would worry about the side effects of drugs.

0 1 2 3 4 5 6 7 8 9 10

OE-M-2. Compliance with prescribed medicine to improve my symptoms of coronary heart disease benefit.

0 1 2 3 4 5 6 7 8 9 10

OE-M-3. Compliance with prescribed medicine will increase the financial burden on me.

0 1 2 3 4 5 6 7 8 9 10
OE-M-4. Compliance with prescribed medicine can prevent acute cardiac event (heart attack, sudden death, etc.)<br>0 1 2 3 4 5 6 7 8 9 10
OE-M-5. Compliance with prescribed medicine will make me healthier.<br>0 1 2 3 4 5 6 7 8 9 10
OE-M-6. Compliance with prescribed medicine makes me feel very comfortable body.<br>0 1 2 3 4 5 6 7 8 9 10
OE-M-7. Compliance with prescribed medicine will affect my image in the minds of others.<br>0 1 2 3 4 5 6 7 8 9 10
OE-M-8. Compliance with prescribed medicine will improve my blood pressure, lipid levels.<br>0 1 2 3 4 5 6 7 8 9 10
OE-M-9. Compliance with prescribed medicine means lower quality of life.<br>0 1 2 3 4 5 6 7 8 9 10
OE-M-10. If compliance with prescribed medicine, people will praise my willpower.<br>0 1 2 3 4 5 6 7 8 9 10
OE-M-11. The total score: ______

MO. Motivation

The following questions are about your level of motivation to change your unhealthy lifestyle. The two ends of the straight line respectively indicate " I don't want at all " (0) and " I want very much (10) ". The higher the value, the more you want to change the unhealthy lifestyle.

MO-1. My current willingness to change unhealthy diets such as high salt and high fat is:<br>0 1 2 3 4 5 6 7 8 9 10

MO-2. My current willingness to change sedentary, irregular exercises is:<br>0 1 2 3 4 5 6 7 8 9 10
MO-3. My current willingness to quit smoking / control smoking is:<br>0 1 2 3 4 5 6 7 8 9 10
MO-4. My current willingness to take medicines that were prescribed by my doctor is:<br>0 1 2 3 4 5 6 7 8 9 10
MO-5. The total score for motivation is: ______

AP. Action Planning

AP-D. Action Planning for Healthy Eating

Do you have an action plan on healthy eating? The two ends of the line indicate "not at all true" (0) and "exactly true (10)" respectively.

AP-D-1. I have planned how to change my eating habits<br>0 1 2 3 4 5 6 7 8 9 10
AP-D-2. I have planned when to change my eating habits<br>0 1 2 3 4 5 6 7 8 9 10
AP-D-3. Total score for dietary action plan: ______

AP-E. Action planning for regular exercise

Do you have an action plan for regular exercise? The two ends of the line indicate "not at all true" (0) and "exactly true (10)" respectively.

AP-E-1. I have planned when to exercise.<br>0 1 2 3 4 5 6 7 8 9 10
AP-E-2. I have planned where to exercise.<br>0 1 2 3 4 5 6 7 8 9 10
AP-E-3. I have planned how to exercise.

0 1 2 3 4 5 6 7 8 9 10
AP-E-4. I have planned how often to exercise.<br>0 1 2 3 4 5 6 7 8 9 10
AP-E-5. I have planned whom to exercise.<br>0 1 2 3 4 5 6 7 8 9 10
AP-E-6. The total score: _______

AP-S. Action planning to quit smoking

Do you have an action plan for quitting smoking? The two ends of the line indicate "not at all true" (0) and "exactly true (10)" respectively.

AP-S-1. I have already planned when to quit smoking<br>0 1 2 3 4 5 6 7 8 9 10
AP-S-2. I have already planned how to quit smoking<br>0 1 2 3 4 5 6 7 8 9 10
AP-S-3. The total score: ________

AP-M. Action plan for taking medication

Do you have an action plan for taking medication regularly? The two ends of the line indicate "not at all true" (0) and "exactly true (10)" respectively.

AP-M-1. I know the name of the drug to be taken<br>0 1 2 3 4 5 6 7 8 9 10
AP-M-2. I know when to take the medicine<br>0 1 2 3 4 5 6 7 8 9 10
AP-M-3. I know the dose of the drug to be taken

0 1 2 3 4 5 6 7 8 9 10

AP-M-4. I remembered how to take medicine

0 1 2 3 4 5 6 7 8 9 10

AP-M-5. The total score: ________

SE. Self-efficacy

SE-D Self-Efficacy of Healthy Eating

How sure are you that you can overcome the following obstacles? The two ends of the line indicate "not at all true" (0) and "exactly true (10)" respectively.

SE-D-1. I can stick to a healthy (low-fat or low-salt) diet, even if I have to learn much about nutrition.

0 1 2 3 4 5 6 7 8 9 10

SE-D-2. I am sure that I can change the unhealthy diet, even if I initially have to watch out in many
situations.

0 1 2 3 4 5 6 7 8 9 10

SE-D-3. I am confident that I can change my unhealthy diet, even if my blood pressure doesn’t improve immediately.

0 1 2 3 4 5 6 7 8 9 10

SE-D-4. I can stick to a healthy (low-fat or low-salt) diet, even if I have to start all over again several times until I succeed.

0 1 2 3 4 5 6 7 8 9 10

SE-D-5. I can stick to a healthy (low-fat or low-salt) diet, even if I initially have to make plans.

0 1 2 3 4 5 6 7 8 9 10

SE-D-6. I can stick to a healthy (low-fat or low-salt) diet, even if initially food doesn’t taste as good.<br>0 1 2 3 4 5 6 7 8 9 10
SE-D-7. I can stick to a healthy (low-fat or low-salt) diet, even if I initially don’t get much support.<br>0 1 2 3 4 5 6 7 8 9 10
SE-D-8. I can stick to a healthy (low-fat or low-salt) diet, even if I takes a long time to get used to it.<br>0 1 2 3 4 5 6 7 8 9 10
SE-D-9. I can stick to a healthy (low-fat or low-salt) diet, even if my cholesterol level doesn't improve immediately.<br>0 1 2 3 4 5 6 7 8 9 10
SE-D-10. I can stick to a healthy (low-fat or low-salt) diet, even if I have worries and troubles.<br>0 1 2 3 4 5 6 7 8 9 10
SE-D-11. I can stick to a healthy (low-fat or low-salt) diet, even if my partner/ my family don’t change their nutrition habits.<br>0 1 2 3 4 5 6 7 8 9 10
SE-D-12. Total dietary self-efficacy score: ________

SE-E. Exercise self-efficacy

SE-EA. Preactional self-efficacy of exercise
It is always hard to get started. How sure are you that you can start exercising regularly? The two ends of the line indicate "not at all true" (0) and "exactly true (10)" respectively.

SE-EA-1. I am sure I can start being physically active immediately, even if I initially have to reconsider my views on physical activity.

0 1 2 3 4 5 6 7 8 9 10
SE-EA-2. I am sure I can start being physically active immediately, the planning for this is very laborious.<br>0 1 2 3 4 5 6 7 8 9 10
SE-EA-3. I am sure I can start being physically active immediately, I have to force myself to start<br>immediately.<br>0 1 2 3 4 5 6 7 8 9 10
SE-EA-4. I am sure I can start being physically active immediately, I have to push myself.<br>0 1 2 3 4 5 6 7 8 9 10
SE-EA-5. Total score of exercise preactional self-efficacy: ________

SE-EC. Coping self-efficacy of exercise

It is important to stay physically active. Are you confident you can manage that?

The two ends of the line indicate "not at all true" (0) and "exactly true (10)" respectively.

SE-EC-1. I am sure I can keep being physically active regularly, even if it takes me long to make it a habit.<br>0 1 2 3 4 5 6 7 8 9 10
SE-EC-2. I am sure I can keep being physically active regularly, even if I am worried and troubled.<br>0 1 2 3 4 5 6 7 8 9 10
SE-EC-3. I am sure I can keep being physically active regularly, even if I don’t see success at once.<br>0 1 2 3 4 5 6 7 8 9 10
SE-EC-4. I am sure I can keep being physically active regularly, even if I am tired.<br>0 1 2 3 4 5 6 7 8 9 10

SE-EC-5. I am sure I can keep being physically active regularly, even if I am stressed out.

0 1 2 3 4 5 6 7 8 9 10

SE-EC-6. I am sure I can keep being physically active regularly, even if I feel tense.

0 1 2 3 4 5 6 7 8 9 10

SE-EC-7. I am sure I can keep being physically active regularly, even if my blood pressure doesn't improve immediately.

0 1 2 3 4 5 6 7 8 9 10

SE-EC-8. I am sure I can keep being physically active regularly, even if I won't get social support for my first attempts.

0 1 2 3 4 5 6 7 8 9 10

SE-EC-9. I am sure I can keep being physically active regularly, even if I have to start all over again several times until I succeed.

0 1 2 3 4 5 6 7 8 9 10

SE-EC-10. I am sure I can keep being physically active regularly, even if my partner/ family isn't physically active.

0 1 2 3 4 5 6 7 8 9 10

SE-EC-11. I am sure I can keep being physically active regularly, even if my cholesterol doesn't improve immediately.

0 1 2 3 4 5 6 7 8 9 10

SE-EC-12. Total score of exercise coping self-efficacy: ________

SE-S. Self-efficacy of coping with smoking cessation

Some conditions make it difficult to quit smoking. How confident are you that you can overcome the following difficulties? The two ends of the line indicate "not at all true" (0) and "exactly true (10)" respectively.

SE-S-1. I can resist smoking, even if I hang out with friends who smoke.

0 1 2 3 4 5 6 7 8 9 10
SE-S-2. I can resist smoking even if I feel tense or nervous.<br>0 1 2 3 4 5 6 7 8 9 10
SE-S-3. I can resist smoking, even though I want to concentrate.<br>0 1 2 3 4 5 6 7 8 9 10
SE-S-4. I can resist smoking, even if I have a strong desire for it.<br>0 1 2 3 4 5 6 7 8 9 10
SE-S-5. I can resist smoking even if I am worried or troubled.<br>0 1 2 3 4 5 6 7 8 9 10
SE-S-6. I can resist smoking, even if I am stressed out.<br>0 1 2 3 4 5 6 7 8 9 10
SE-S-7. I can resist smoking, even if I am around friends or colleagues who smoke.<br>0 1 2 3 4 5 6 7 8 9 10
SE-S-8. I can resist smoking, even when I get little support during my first attempts to quit.<br>0 1 2 3 4 5 6 7 8 9 10
SE-S-9. I can resist smoking, even if I have to start all over again several times until I succeed.<br>0 1 2 3 4 5 6 7 8 9 10
SE-S-10. I can resist smoking, even if I let myself be seduced some time.<br>0 1 2 3 4 5 6 7 8 9 10
SE-S-11. I can resist smoking even if my partner/ family don’t quit smoking.<br>0 1 2 3 4 5 6 7 8 9 10
SE-S-12. I can resist smoking, even if I want to push myself.<br>0 1 2 3 4 5 6 7 8 9 10

SE-S-13. Total self-efficacy score for quitting smoking: ________

SE-M. Self-efficacy of taking medicine regularly

Certain obstacles make it difficult to change a person's medication compliance. How confident are you that you can overcome the following difficulties? The two ends of the line indicate "not at all true" (0) and "exactly true (10)" respectively.

SE-M-1. I can stick to taking medication, even if I have a bad memory.

0 1 2 3 4 5 6 7 8 9 10

SE-M-2. I can stick to taking medication, even when I am traveling or having a meeting.

0 1 2 3 4 5 6 7 8 9 10

SE-M-3. I can stick to taking medication, even if my health indicators (blood sugar, blood pressure, blood lipids, etc.) do not improve immediately.

0 1 2 3 4 5 6 7 8 9 10

SE-M- 4. I can stick to taking medication, prescribed medication, even if it is difficult at the beginning.

0 1 2 3 4 5 6 7 8 9 10

SE-M-5. I can stick to taking medication, even beginning I did not get much support.

0 1 2 3 4 5 6 7 8 9 10

SE-M-6. I can stick to taking medication, even if I take a long time to adapt.

0 1 2 3 4 5 6 7 8 9 10

SE-M-7. I can stick to taking medication, even if I have concerns and worries (such as fear of side effects, the economic burden).

0 1 2 3 4 5 6 7 8 9 10

SE-M-8. The total score for medication self-efficacy: ________

VO. Volition

The following questions are about your level of volition to exercise regularly, eat a healthy diet, quit smoking, and regularly take the medication prescribed by your doctor. The two ends of the line indicate " very low " (0) and " very high (10) " respectively. The higher the value, the higher the level of willpower you think you have.

VO-1. Eating a low-salt and low-fat diet every day, my volition is:

0 1 2 3 4 5 6 7 8 9 10

VO-2. Taking exercise 3-5 times a week for at least 30 minutes each time, my volition is:

0 1 2 3 4 5 6 7 8 9 10

VO-3. Do not smoke every day, my volition is:

0 1 2 3 4 5 6 7 8 9 10

VO-4. Take the medicine every day according to the doctor's advice, my volition is:

0 1 2 3 4 5 6 7 8 9 10

VO-5. Total score of volition: ________

CP. Coping Planning

CP-D. Coping planning for diet

Most people would like to further improve their nutrition habits by taking in less salt and fat. How about you? The two ends of the line indicate "not at all true" (0) and "exactly true (10)" respectively.

CP-D-1. I already have concrete plans when to especially watch out in order to maintain my new nutrition habits.

0 1 2 3 4 5 6 7 8 9 10

CP-D-2. I already have concrete plans what to do in difficult situations in order to stick to my intentions.<br>0 1 2 3 4 5 6 7 8 9 10
CP-D-3. I already have concrete plans how to deal with relapses.<br>0 1 2 3 4 5 6 7 8 9 10
CP-D-4. The total score of the diet copping planning: ________

CP-E. Copping planning for exercise

Do you have a coping plan for regular exercise? The two ends of the line indicate "not at all true" (0) and "exactly true (10)" respectively.

CP-E-1. I already have concrete plans what to do if something intervenes.<br>0 1 2 3 4 5 6 7 8 9 10
CP-E-2. I already have concrete plans what to do if I miss an exercise session.<br>0 1 2 3 4 5 6 7 8 9 10
CP-E-3. I already have concrete plans what to do in difficult situations in order to stick to my intentions.<br>0 1 2 3 4 5 6 7 8 9 10
CP-E-4. I already have concrete plans when to especially watch out in order to stay committed.<br>0 1 2 3 4 5 6 7 8 9 10
CP-E-5. The total score of copping planning for exercise: ________

CP-S. Copping planning for smoking cessation

Do you have a coping plan for smoking cessation? The two ends of the line indicate "not at all true" (0) and "exactly true (10)" respectively.

CP-S-1. I already have concrete plans how to deal with relapses into my old habits.

0 1 2 3 4 5 6 7 8 9 10

CP-S-2. I already have concrete plans what to do in difficult situations to stick to my intentions.

0 1 2 3 4 5 6 7 8 9 10

CP-S-3. I already have concrete plans when to especially watch out in order not to reach again for a cigarette.

0 1 2 3 4 5 6 7 8 9 10

CP-S-4. Total score of copping planning for smoking cessation: ________

CP-M. Copping planning for taking medication

Do you have a copping plan to take medication? The two ends of the line indicate "not at all true" (0) and "exactly true (10)" respectively.

CP-M-1. I already have concrete plans when I need to take care in case of forgetting to take the medicine.

0 1 2 3 4 5 6 7 8 9 10

CP-M-2. I already have concrete plans how to deal with when occasionally forgetting to take the medicine.

0 1 2 3 4 5 6 7 8 9 10

CP-M-3. I already have concrete plans to adhere to taking medicine when I have adverse difficulties.

0 1 2 3 4 5 6 7 8 9 10

CP-M-4. I already have concrete plans how to prevent the situation that causes the interruption of taking medication.

0 1 2 3 4 5 6 7 8 9 10

CP-M-5. The total score of the copping planning for taking medication: ________

EP. Efficacy Perception

Adopting a healthy lifestyle and taking medication as prescribed by a doctor, how do you perceive the improvement of health indicators? The two ends of the straight line indicate "almost no improvement" (0) and "very much improvement (10)" respectively. The higher the value, the greater the improvement you feel.

EP-1. The degree to which I perceive my blood pressure improvement (or the degree to which it remains at an ideal level) is:<br>0 1 2 3 4 5 6 7 8 9 10
EP-2. The degree to which I perceive the improvement (or the degree to which it is maintained at an ideal level) of my blood lipids is:<br>0 1 2 3 4 5 6 7 8 9 10
EP-3. The degree to which I perceive my blood sugar improvement (or the degree to which it remains at an ideal level) is:<br>0 1 2 3 4 5 6 7 8 9 10
EP-4. The degree to which I perceive the improvement of my weight (or the degree to which it is maintained at an ideal level) is:<br>0 1 2 3 4 5 6 7 8 9 10
EP-5. I perceive the degree of improvement of my vascular plaque:<br>0 1 2 3 4 5 6 7 8 9 10
EP-6. I perceive the degree of improvement in my overall health:<br>0 1 2 3 4 5 6 7 8 9 10
EP-7. Total score for efficacy perception: ________

BE. Behavioral Enjoyment

Behavioral enjoyment of healthy eating

Please rate how you feel when you eating healthily? The two ends of the line indicate "I enjoy it" (0) and "I hate it (10)" respectively.

BE-D-1. I enjoy it.<br>0 1 2 3 4 5 6 7 8 9 10
BE-D-2. I feel bored.<br>0 1 2 3 4 5 6 7 8 9 10
BE-D-3. I don’t like it.<br>0 1 2 3 4 5 6 7 8 9 10
BE-D-4. I find it pleasurable.<br>0 1 2 3 4 5 6 7 8 9 10
BE-D-5. It’s no fun at all.<br>0 1 2 3 4 5 6 7 8 9 10
BE-D-6. I find it energizing.<br>0 1 2 3 4 5 6 7 8 9 10
BE-D-7. It makes me depressed.<br>0 1 2 3 4 5 6 7 8 9 10
BE-D-8. It’s very pleasant.<br>0 1 2 3 4 5 6 7 8 9 10
BE-D-9. I feel good physically while doing it.<br>0 1 2 3 4 5 6 7 8 9 10
BE-D-10. It’s very invigorating.<br>0 1 2 3 4 5 6 7 8 9 10
BE-D-11. I am very frustrated.

0 1 2 3 4 5 6 7 8 9 10

BE-D-12. It’s very exhilarating.

0 1 2 3 4 5 6 7 8 9 10

BE-D-13. It’s not at all stimulating.

0 1 2 3 4 5 6 7 8 9 10

BE-D-14. It gives me a strong sense of accomplishment.

0 1 2 3 4 5 6 7 8 9 10

BE-D-15. It’s very refreshing.

0 1 2 3 4 5 6 7 8 9 10

BE-D-16. I felt as though I would rather be doing something else.

0 1 2 3 4 5 6 7 8 9 10

BE-D-17. The total score: ________

BE-E. Behavioral enjoyment of exercise

Please rate how you feel at the moment about the physical activity you have been doing? The two ends of the line indicate "I enjoy it" (0) and "I hate it (10)" respectively.

BE-E-1. I enjoy it.

0 1 2 3 4 5 6 7 8 9 10

BE-E-2. I feel bored.

0 1 2 3 4 5 6 7 8 9 10

BE-E-3. I don’t like it.

0 1 2 3 4 5 6 7 8 9 10

BE-E-4. I find it pleasurable.

0 1 2 3 4 5 6 7 8 9 10

BE-E-5. It's no fun at all.<br>0 1 2 3 4 5 6 7 8 9 10
BE-E-6. I find it energizing.<br>0 1 2 3 4 5 6 7 8 9 10
BE-E-7. It makes me depressed.<br>0 1 2 3 4 5 6 7 8 9 10
BE-E-8. It's very pleasant.<br>0 1 2 3 4 5 6 7 8 9 10
BE-E-9. I feel good physically while doing it.<br>0 1 2 3 4 5 6 7 8 9 10
BE-E-10. It's very invigorating.<br>0 1 2 3 4 5 6 7 8 9 10
BE-E-11. I am very frustrated.<br>0 1 2 3 4 5 6 7 8 9 10
BE-E-12. It's very exhilarating.<br>0 1 2 3 4 5 6 7 8 9 10
BE-E-13. It's not at all stimulating.<br>0 1 2 3 4 5 6 7 8 9 10
BE-E-14. It gives me a strong sense of accomplishment.<br>0 1 2 3 4 5 6 7 8 9 10
BE-E-15. It's very refreshing.<br>0 1 2 3 4 5 6 7 8 9 10
BE-E-16. I felt as though I would rather be doing something else.<br>0 1 2 3 4 5 6 7 8 9 10

BE-E-17. The total score: ________

BE-E. Behavioral enjoyment of quitting smoking

Please rate how you feel at the moment about quitting smoking? The two ends of the line indicate "I enjoy it" (0) and "I hate it (10)" respectively.

BE-S-1. I enjoy it.

0 1 2 3 4 5 6 7 8 9 10

BE-S-2. I feel bored.

0 1 2 3 4 5 6 7 8 9 10

BE-S-3. I don’t like it.

0 1 2 3 4 5 6 7 8 9 10

BE-S-4. I find it pleasurable.

0 1 2 3 4 5 6 7 8 9 10

BE-S-5. It’s no fun at all.

0 1 2 3 4 5 6 7 8 9 10

BE-S-6. I find it energizing.

0 1 2 3 4 5 6 7 8 9 10

BE-S-7. It makes me depressed.

0 1 2 3 4 5 6 7 8 9 10

BE-S-8. It’s very pleasant.

0 1 2 3 4 5 6 7 8 9 10

BE-S-9. I feel good physically while doing it.

0 1 2 3 4 5 6 7 8 9 10

BE-S-10. It’s very invigorating.

0 1 2 3 4 5 6 7 8 9 10

BE-S-11. I am very frustrated.

0 1 2 3 4 5 6 7 8 9 10
BE-S-12. It’s very exhilarating.<br>0 1 2 3 4 5 6 7 8 9 10
BE-S-13. It’s not at all stimulating.<br>0 1 2 3 4 5 6 7 8 9 10
BE-S-14. It gives me a strong sense of accomplishment.<br>0 1 2 3 4 5 6 7 8 9 10
BE-S-15. It’s very refreshing.<br>0 1 2 3 4 5 6 7 8 9 10
BE-S-16. I felt as though I would rather be doing something else.<br>0 1 2 3 4 5 6 7 8 9 10
BE-S-17. The total score: ________

BE-E. Behavioral enjoyment of taking medicine regularly

Please rate how you feel at the moment about taking medicine regularly? The two ends of the line indicate "I enjoy it" (0) and "I hate it (10)" respectively.

BE-M-1. I enjoy it.<br>0 1 2 3 4 5 6 7 8 9 10
BE-M-2. I feel bored.<br>0 1 2 3 4 5 6 7 8 9 10
BE-M-3. I don’t like it.<br>0 1 2 3 4 5 6 7 8 9 10
BE-M-4. I find it pleasurable.<br>0 1 2 3 4 5 6 7 8 9 10
BE-M-5. It’s no fun at all.

0 1 2 3 4 5 6 7 8 9 10
BE-M-6. I find it energizing.<br>0 1 2 3 4 5 6 7 8 9 10
BE-M-7. It makes me depressed.<br>0 1 2 3 4 5 6 7 8 9 10
BE-M-8. It's very pleasant.<br>0 1 2 3 4 5 6 7 8 9 10
BE-M-9. I feel good physically while doing it.<br>0 1 2 3 4 5 6 7 8 9 10
BE-M-10. It's very invigorating.<br>0 1 2 3 4 5 6 7 8 9 10
BE-M-11. I am very frustrated.<br>0 1 2 3 4 5 6 7 8 9 10
BE-M-12. It's very exhilarating.<br>0 1 2 3 4 5 6 7 8 9 10
BE-M-13. It's not at all stimulating.<br>0 1 2 3 4 5 6 7 8 9 10
BE-M-14. It gives me a strong sense of accomplishment.<br>0 1 2 3 4 5 6 7 8 9 10
BE-M-15. It's very refreshing.<br>0 1 2 3 4 5 6 7 8 9 10
BE-M-16. I felt as though I would rather be doing something else.<br>0 1 2 3 4 5 6 7 8 9 10
BE-M-17. The total score: ________

SS. Social Support

The following questions are used to reflect the support you have received in society. The two ends of the line indicate "not at all degree" (0) and " strongly degree (10)" respectively.

SS-1. When I have a problem, some people (leaders, relatives, colleagues) will help me.<br>0 1 2 3 4 5 6 7 8 9 10
SS-2. I can share happiness and sadness with some people (leaders, relatives, colleagues)<br>0 1 2 3 4 5 6 7 8 9 10
SS-3. My family can help me concretely.<br>0 1 2 3 4 5 6 7 8 9 10
SS-4. I can get emotional help and support from my family when I need it.<br>0 1 2 3 4 5 6 7 8 9 10
SS-5. Some people (leaders, relatives, colleagues) are the real source of comfort when I have difficulties.<br>0 1 2 3 4 5 6 7 8 9 10
SS-6. My friends can really help me.<br>0 1 2 3 4 5 6 7 8 9 10
SS-10. I can rely on my friends when I am in trouble.<br>0 1 2 3 4 5 6 7 8 9 10
SS-8. I can talk about my problems with my family.<br>0 1 2 3 4 5 6 7 8 9 10
SS-9. My friends can share happiness and sadness with me.

0 1 2 3 4 5 6 7 8 9 10
SS-10. There are certain people (leaders, relatives, colleagues) in my life who care about my feelings.<br>0 1 2 3 4 5 6 7 8 9 10
SS-11. My family is willing to assist me in making various decisions.<br>0 1 2 3 4 5 6 7 8 9 10
SS-12. I can discuss my problems with my friends.<br>0 1 2 3 4 5 6 7 8 9 10
SS-13. Total score for social support: ________

Understanding of the Importance of Healthy Lifestyles

The following questions are about your understanding of the importance of exercising regularly, eating a healthy diet, quitting smoking, and taking regular medication prescribed by your doctor. The two ends of the line indicate "not important at all " (0) and "very important (10)", respectively. The higher the value, the more important you think.

IM-1. Eating a low-salt and low-fat diet every day is:<br>0 1 2 3 4 5 6 7 8 9 10
IM-2. Taking exercise 3-5 times a week for at least 30 minutes each time is:<br>0 1 2 3 4 5 6 7 8 9 10
IM-3. Do not smoke every day is:<br>0 1 2 3 4 5 6 7 8 9 10
IM-4. Taking the medicine daily according to the doctor's advice is:<br>0 1 2 3 4 5 6 7 8 9 10
IM-5. The total score for the understanding of the importance of a healthy lifestyle is: ________

Coronary Artery Disease Education Questionnaire (CADE - Q)

Q1	Coronary artery disease (CAD) is:
	a) A disease of the arteries of the heart that occurs in older age in people with high cholesterol and who smoke.
	b) A disease of the arteries of the heart that starts silently at a young age, is influenced by poor life style habits, has a genetic component, and involves inflammation in the arteries.
	c) A disease of the heart's arteries related to older age and that leads to memory Impairment.
	d) I don't know.
Q2	Which factors have the most influence on the risk of myocardial infarction?
	a) Drinking small amounts of alcoholic beverages.
	b) Environment factors (such as weather) and socioeconomic factors (such as monthly family income).
	c) Smoking, high levels of blood cholesterol (dyslipidemia), and hypertension.
	d) I don't know.
Q3	Which description below is a typical symptom of CAD?
	a) Headache after meals.
	b) Chest pain or discomfort during physical activity.
	c) Chest pain or discomfort, at rest or during physical activity, which can also be felt in the arm and/or back and/or neck.

	d) I don't know.
Q4	Which of the following statements is most accurate regarding our understanding of CAD?
	a) The CAD is related to blockage of the arteries that supply blood to the heart caused by the formation of atherosclerotic plaques (fat deposit on the artery walls), that can cause angina (chest pain).
	b) Acute myocardial infarction (MI) is the only manifestation of CAD.
	c) The presence of chest pain is suggestive of a diagnosis of CAD.
	d) I don't know.
Q5	The best time of the day for people with coronary disease to carry out their prescribed exercise is:
	a) In the afternoon or evening, because the early morning is the time of day with the highest risk of a heart attack.
	b) Never, because exercise is considered too risky for people with CAD.
	c) Any time, because the benefits of exercise outweigh the risks at any time of day.
	d) I don't know.
Q6	Of the investigations listed below, which ones provide the most precise information about the diagnosis and prognosis of CAD?
	a) X-ray and magnetic resonance imaging of the chest.
	b) Exercise treadmill test (stress test) and cardiac catheterization (angiogram).
	c) Electrocardiogram (EKG) at rest and a clinical history.

	d) I don't know.
Q7	Which of the following statements about the management of blood cholesterol levels is most accurate?
	a) Physical exercise and diet are enough to lower cholesterol to target levels after a heart attack.
	b) Physical exercise and diet should be followed regularly and when necessary, a medication such as a "statin" may be required
	c) There is no treatment because high cholesterol levels are genetically inherited and can't be changed.
	d) I don't know.
Q8	Which of the following statements about the use of 'nitroglycerin' is most accurate?
	a) They are a class of medications that can be administered to improve coronary blood flow and can be given either continuously (such as in a tablet or patch) or used sublingually (under the tongue as a spray or small tablet) in situations of acute chest pain.
	b) They are medications given only by the sublingual route in emergency situations to relieve chest pain.
	c) They are medicines used to decrease blood pressure and bad cholesterol (LDL) in patients with cardiac problems.
	d) I don't know.
Q9	Which of the following dietary components is usually recommended to persons with CAD?
	a) A diet with reduced salt, low fat and rich in fiber.
	b) A diet based on whole grains, vegetables, fish, extra virgin olive oil and nuts.

	c) An unrestricted diet, because diet is not a relevant factor.
	d) I don't know.
Q10	Which values for LDL cholesterol and HDL cholesterol are the optimal targets persons with established CAD (values in mmol/l)?
	a) LDL less than 2.0 and HDL greater than 1.2.
	b) LDL 2.0 to 2.5 and HDL greater than 1.0.
	c) LDL greater than 3.0 and HDL less than 1.0.
	d) I don't know.
Q11	Under which of the following conditions would you avoid carrying out your usual physical exercise?
	a) If you had a recent heart attack (for example 8 weeks ago).
	b) If you have a bad infection today (for example a really bad 'flu').
	c) If your blood pressure is moderately elevated (for example 150/90).
	d) I don't know.
Q12	While walking, if you experience a new episode of severe chest discomfort that you think that is angina, you should:
	a) Drive your car directly to the hospital to seek medical care.
	b) Try to relax, wait for the pain to improve, and then seek medical attention.

	c) Stop your walk and sit, take a sublingual nitroglycerin, and seek medical care if the pain does not subside. Call your doctor to let him or her know what has happened.
	d) I don’t know.
Q13	Based on your knowledge about physical exercise and CAD, choose the most appropriate statement below:
	a) Physical exercise should never be practiced by patients with coronary artery disease because of high risk of death.
	b) Physical exercise is a fundamental part of the treatment plan, because it helps to control risk factors, prolongs survival and enhances quality of life.
	c) Physical activity should be included in the treatment plan only when patients are fully recovered from their heart event.
	d) I don’t know.
Q14	Guidelines for physical activity for people with coronary disease should be based upon which of the following:
	a) The exercise prescription should be individually devised based on an exercise stress test and respect the person’s abilities and disabilities.
	b) Start at a low level to moderate level and build up gradually.
	c) Be the same for all persons of the same gender and age, because these groups have the same physical ability and risk.
	d) I don’t know.
Q15	Which of the following favorable physiological and bodily changes resulting from regular physical exercise are most important to long-term cardiac health?

	a) Blood vessel function improvement, growth of new blood vessels, and even a possible regression (shrinking) of atherosclerotic plaque.
	b) Resting heart rate decrease, more forceful heart beat, and lipid profile improvement.
	c) Blood pressure increase, higher heart rates, and higher triglyceride levels.
	d) I don't know.
Q16	Which of the following statements best describes the pattern for exercise activity in persons recovering from a heart event:
	a) At any place, daily duration of about 30 minutes, which can be cumulative (10 minutes in the morning, 10 minutes at noon and 10 minutes at night).
	b) In an appropriate setting, with periodic monitoring by qualified professionals, with the goal of achievement of self-sufficiency.
	c) In a hospital environment only.
	d) I don't know.
Q17	Which of the following statements is the most appropriate guidance around levels of blood pressure levels in persons with CAD:
	a) It doesn't matter whether blood pressure is normal or high because it does not have any long-term health effects.
	b) A value of less than 140/90 mmHg is considered normal.
	c) An optimal blood pressure is 120/80 mmHg.
	d) I don't know.

Q18	Which of the statements below regarding psychological stress is most correct?
	a) It is one of the important risk factors for AMI (acute myocardial infarction).
	b) Stress is related to the presence of anxiety and depression.
	c) It has no impact on heart disease, since atherosclerosis is a completely physical process and is not related to psychological factors.
	d) I don’t know.
Q19	Which interventions can extend and improve a patient’s quality of life for persons recovering from a cardiac event?
	a) Lifestyle changes + medical treatments + in some cases surgical intervention.
	b) Medication + in some cases surgical intervention.
	c) Prolonged bed rest.
	d) I don’t know.
